# Multiple transatlantic incursions of highly pathogenic avian influenza clade 2.3.4.4b A(H5N5) virus into North America and spillover to mammals

**DOI:** 10.1016/j.celrep.2024.114479

**Published:** 2024-07-13

**Authors:** Cassidy N.G. Erdelyan, Ahmed Kandeil, Anthony V. Signore, Megan E.B. Jones, Peter Vogel, Konstantin Andreev, Cathrine Arnason Bøe, Britt Gjerset, Tamiru N. Alkie, Carmencita Yason, Tamiko Hisanaga, Daniel Sullivan, Oliver Lung, Laura Bourque, Ifeoluwa Ayilara, Lemarie Pama, Trushar Jeevan, John Franks, Jeremy C. Jones, Jon P. Seiler, Lance Miller, Samira Mubareka, Richard J. Webby, Yohannes Berhane

**Affiliations:** 1 National Centre for Foreign Animal Disease, Winnipeg, MB R3E 3M4, Canada; 2 Department of Pathology and Host-Microbe Interactions, St. Jude Children’s Research Hospital, Memphis, TN 38105, USA; 3 Center of Scientific Excellence for Influenza Viruses, National Research Centre, Giza, 12622, Egypt; 4 Canadian Wildlife Health Cooperative, Atlantic Region, Charlottetown, PEI C1A 4P3, Canada; 5 Comparative Pathology Core, St. Jude Children’s Research Hospital, Memphis, TN 38105, USA; 6 Norwegian Veterinary Institute, 1433 Ås, Norway; 7 Atlantic Veterinary College, University of Prince Edward Island, Charlottetown, PEI C1A 4P3, Canada; 8 Department of Biological Sciences, University of Manitoba, Winnipeg, MB R3T 2M5, Canada; 9 Sunnybrook Research Institute, Toronto, ON M4N 3M5, Canada; 10 Department of Laboratory Medicine and Pathobiology, Temerty Faculty of Medicine, University of Toronto, Toronto, ON M5S 1A1, Canada; 11 Department of Microbiology, Immunology, and Biochemistry, University of Tennessee Health Science Center, Memphis, TN 38105, USA; 12 Department of Animal Science, University of Manitoba, Winnipeg, MB R3T 2N2, Canada; 13 Department of Veterinary Pathology, Western College of Veterinary Medicine, University of Saskatchewan, Saskatoon, SK S7N 5B4, Canada; 14 These authors contributed equally; 15 Lead contact

## Abstract

Highly pathogenic avian influenza (HPAI) viruses have spread at an unprecedented scale, leading to mass mortalities in birds and mammals. In 2023, a transatlantic incursion of HPAI A(H5N5) viruses into North America was detected, followed shortly thereafter by a mammalian detection. As these A(H5N5) viruses were similar to contemporary viruses described in Eurasia, the transatlantic spread of A(H5N5) viruses was most likely facilitated by pelagic seabirds. Some of the Canadian A(H5N5) viruses from birds and mammals possessed the PB2-E627K substitution known to facilitate adaptation to mammals. Ferrets inoculated with A(H5N5) viruses showed rapid, severe disease onset, with some evidence of direct contact transmission. However, these viruses have maintained receptor binding traits of avian influenza viruses and were susceptible to oseltamivir and zanamivir. Understanding the factors influencing the virulence and transmission of A(H5N5) in migratory birds and mammals is critical to minimize impacts on wildlife and public health.

## In brief

Erdelyan et al. reveal that seabirds likely facilitated the spread of A(H5N5) viruses from Europe to Canada. All viral segments were Eurasian. Hemagglutinin contained substitutions not present in Canadian A(H5N1) viruses. These viruses remain susceptible to neuraminidase inhibitors. In ferrets, the virus caused rapid mortality, with some transmission to contacts.

## Graphical abstract

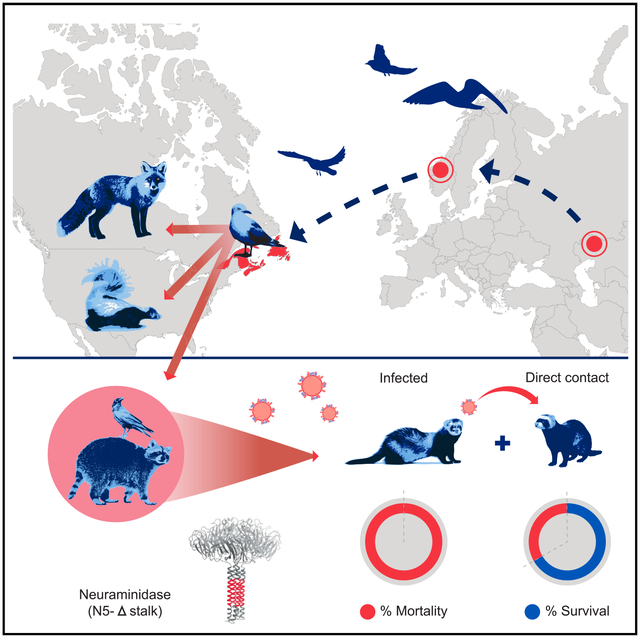

## INTRODUCTION

The influenza A virus (IAV) genome comprises eight discrete segments of single-stranded negative-sense RNA that encode at least 11 proteins. These include polymerase components (segments PB2, PB1, and PA), nucleoprotein (segment NP), matrix proteins (segment M), non-structural proteins (segment NS), and surface glycoproteins (segments HA and NA). The two structural surface glycoproteins, hemagglutinin (HA) and neuraminidase (NA), are associated with antigenicity and pathogenicity in various hosts. In avian species, 16 HA and 9 NA subtypes have been identified, with aquatic waterfowl having the most diversity.^1^ Typically, these avian viruses exist as low pathogenicity avian influenza (LPAI) viruses. However, viruses of the H5 and H7 subtypes can become highly pathogenic avian influenza (HPAI)^2^ through the acquisition of multiple basic amino acids in the HA cleavage site motif, which typically occurs following spillover infections in gallinaceous poultry species.

Waterfowl (Anseriformes) and shorebirds (Charadriiformes) serve as reservoir hosts for LPAI viruses and typically do not display clinical signs upon infection.^3,4^ The distribution of IAV subtypes varies across different species of migratory birds,^5^ and these birds can be simultaneously infected with more than one virus, allowing viral evolution through genetic reassortment. Dense congregations of wild birds harboring different IAVs at breeding or overwintering sites increase contact transmission and the likelihood of generating reassortants.^6^ Reassortment events select for the most fit gene combinations and lead to a transient increase in putatively adaptive amino acid substitutions, which may generate IAVs that are adapted to new hosts and can spread readily.^7,8^ IAVs preferentially replicate in the avian host’s intestinal tract, resulting in the shedding of virions^9^ into the environment and potential infection of susceptible species.

HPAI A(H5N1) A/Goose/Guangdong/1/96 (GsGd) viruses were detected in 1996 from domestic geese in southeast Asia and have changed the landscape of avian influenza virus (AIV) epidemiology and evolution. GsGd-lineage viruses^10,11^ caused mass mortality in migratory waterfowl in April 2005 at Lake Qinghai, China,^12,13^ highlighting the role of migratory birds in their spread.^13–16^ The GsGd-lineage viruses subsequently spread to over 80 countries^17^ throughout Asia, Europe, Africa,^15,18^ and also North America in 2014.^19^

The GsGd-lineage clades 2.2,^12^ 2.3.2,^20^ and 2.3.4.4^16^ have been reported to cause global epidemics in both wild birds and poultry. Importantly, the clade 2.3.4.4 H5 viruses that emerged in 2008^21^ contained HA mutations, allowing reassortment with numerous NAs, creating multiple A(H5Nx) subtype viruses.^22–24^ While clade 2.3.4.4 A(H5Nx) viruses are dispersed globally and remain entrenched in Asia, Africa, Europe, and the Middle East, their occurrence in the Americas is a recent phenomenon.^25^ As of May 2023, more than 870 human infections with GsGd-lineage A(H5N1) have been documented across 23 countries, resulting in over 450 fatalities.^26^ So far, only 7 human deaths have been attributed to clade 2.3.4.4b.^27^

The emergence of clade 2.3.4.4b A(H5N8) HPAI viruses in 2016–2017 in Europe inflicted major and widespread outbreaks in poultry and wild birds. This virus became the predominant subtype in Europe^28^ and parts of Asia^29^ until 2020,^25^ when a descendant A(H5N1) reassortant was detected in the Netherlands that eventually dominated globally.^25,28,29^ Almost 200 million poultry died or were destroyed globally from 2020 to November 2022 due to clade 2.3.4.4b A(H5Nx) viruses, approximately the same number as from 2005 to 2019. Of the 200 million poultry deaths, 54 million were from North America^30,31^ following the virus’ introduction in November 2021. The early North American 2.3.4.4b A(H5N1) viruses contained a fully Eurasian (EA)-lineage genome and were detected in Atlantic Canada and later throughout North^32,33^ and South America.^34^ Only Oceania has yet to report any cases of 2.3.4.4b.^35,36^ The geographic movement of A(H5Nx) IAVs is important to track, since their widespread prevalence and incursions to new localities may pose a considerable threat to economic and food security, as well as human health.

The current outbreak of HPAI in the Americas is dominated by A(H5N1) viruses, with detections of A(H5N5) viruses rare.^37^ Clade 2.3.4.4 viruses are prone to frequent reassortment,^38,39^ a mechanism for rapid virus evolution.^40^ Recently, clade 2.3.4.4b A(H5N5) viruses were detected in Russia, Romania, Bulgaria, and Norway, some containing an unreported NA stalk deletion.^41^ Iceland is a known staging ground for IAV-infected gulls and other water-associated birds^42,43^ and is implicated in the possible spread of clade 2.3.4.4b from Europe to North America,^44,45^ connecting the east Atlantic and north Atlantic American Flyways.^45^ Greenland could also play a similar role in the spread of IAV,^32,46^ along with Svalbard, a Norwegian high arctic archipelago.^32,47,48^

In the present study, we describe the incursion of a fully EA clade 2.3.4.4b A(H5N5) virus into North America, most likely through the movement of seabirds across the Atlantic Flyway. We also present cases of A(H5N5) in multiple species of mesocarnivores—small mammals, like raccoons, skunks, and foxes. We examine viral evolutionary relationships, possible transatlantic routes of incursion from Eurasia to North America, possible intermediate hosts, *in vitro* antiviral susceptibility, and the virulence exhibited by A(H5N5) in a ferret model.

## RESULTS

### Description and location of A(H5N5)-affected animal species

A total of 41 cases of A(H5N5) have been detected in Canadian wildlife as of June 2023, all occurring in the Maritime provinces of Prince Edward Island (PEI), New Brunswick (NB), and Nova Scotia (NS), on the east coast of Canada. The earliest Canadian detection of A(H5N5) was in an American crow (A/American_Crow/PEI/FAV-0035-6/2023) that was found dead on January 9, 2023 in Summerside, PEI. Subsequently, A(H5N5) viruses were detected on January 31 in three additional American crows (A/American_Crow/PEI/FAV-0068-3/2023, A/American_Crow/PEI/FAV-0068-8/2023, and A/American_Crow/PEI/FAV-00689/2023) that were also found dead in Summerside less than 100 m from the earliest case. Five infected mammals (four raccoons and one skunk) were found on PEI, while two foxes were found in NS, near Halifax. The two initial raccoon samples (A/Raccoon/PEI/FAV-0193-1/2023 and A/Raccoon/PEI/FAV-0199-1/2023) were located in Sea View, PEI approximately 20 km away from the earliest Canadian case of A(H5N5). The two raccoons were found dead on April 3 and 29, 1.4 km apart.

**Figure 1.**
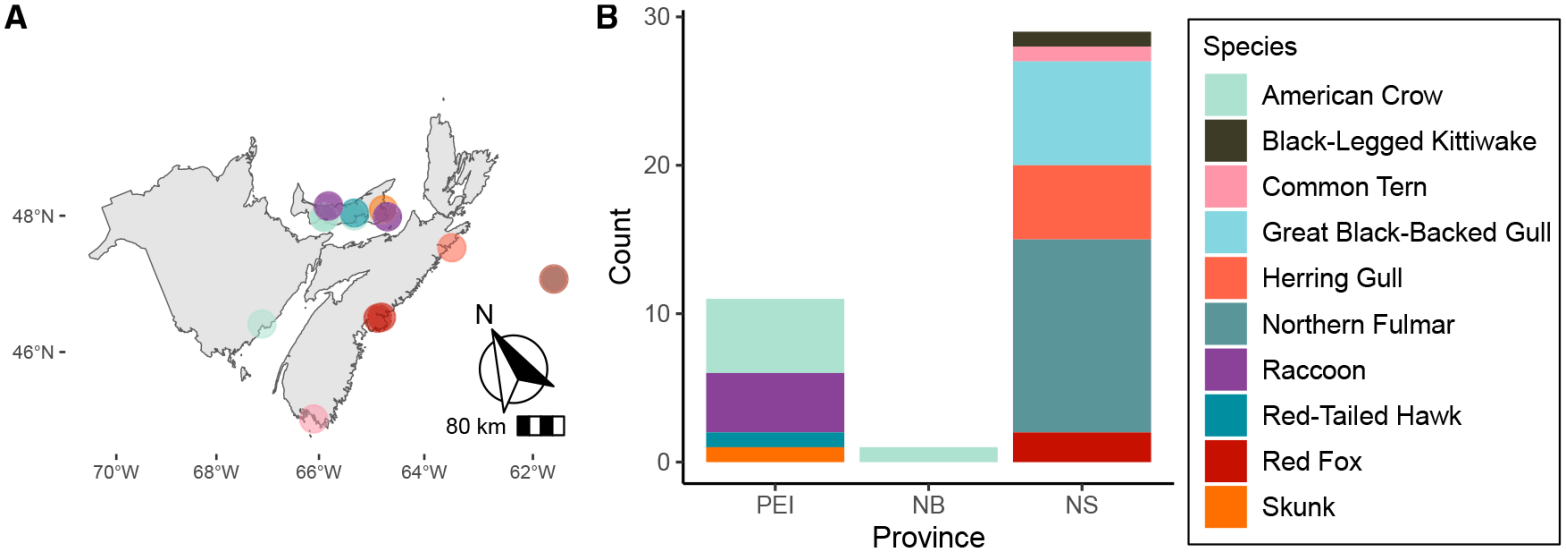
A(H5N5) cases in the Atlantic provinces of Canada (*n* = 41) (A) Locations where animals were collected. (B) Type of wild bird and mammalian species that virus was detected in.

Samples were collected from a black-legged kittiwake, great black-backed gulls (GBBGs), northern fulmars, and herring gulls (*n* = 1, 7, 13, and 4, respectively) from Sable Island, NS (located ~290 km offshore from Halifax) between February 16 and April 30, 2023 (Figure 1). One additional herring gull with A(H5N5) was found dead at Country Island, NS on June 2, 2023. Country Island is 5 km off the coast of mainland NS, one of the closest points to Sable Island.

Almost all animals that were diagnosed with A(H5N5) virus infection were found dead, except for a red fox pup near Halifax, NS that was found sick, displaying neurological signs such as disorientation, and barely walking. This fox pup died before arrival at a wildlife rehabilitation center. A(H5N5) viruses were isolated from three raccoon brain samples. Species of animals infected with A(H5N5) and their geographic location are summarized in Figure 1.

### Mutations associated with mammalian adaptation

A(H5N5) viruses isolated from both mammals and birds had mutations associated with mammalian adaptation. Viruses from two mammals (A/Raccoon/PEI/FAV-0199-1/2023 and A/Striped_Skunk/PEI/FAV-0210-1/2023) and two gulls (A/Great_Black-Backed_Gull/NS/FAV-0263-8/2023 and A/Herring_Gull/NS/FAV-0263-6/2023) had the mammalian adaptive E627K mutation in the polymerase basic protein (PB2). PB2-E627K yields more efficient IAV replication in mammalian cells. Viruses from the infected mammals appeared to have acquired the E627K mutations independently as they were phylogenetically distinct from one another and clustered with other A(H5N5) viruses containing PB2-E627, while the two gulls most likely acquired it from a common source due to sequence similarity (Figure S1).

Some viruses from Sable Island had mutations not seen in other A(H5N5) isolates. A/Black-Legged_Kittiwake/NS/FAV-0264-11/2023 had two mutations previously associated with increased virulence in mammals, PB2-I292V and PB1-F2-N66S. While no other viruses appeared to have descended from this specific virus, it was more closely related to the A(H5N5) from the only skunk sample (A/Striped_Skunk/PEI/FAV-0210-1/2023).

Other experimentally supported amino acid substitutions associated with adaptation in birds and mammals are summarized in Table S1.

### Phylogenetic analysis

**Figure 2.**
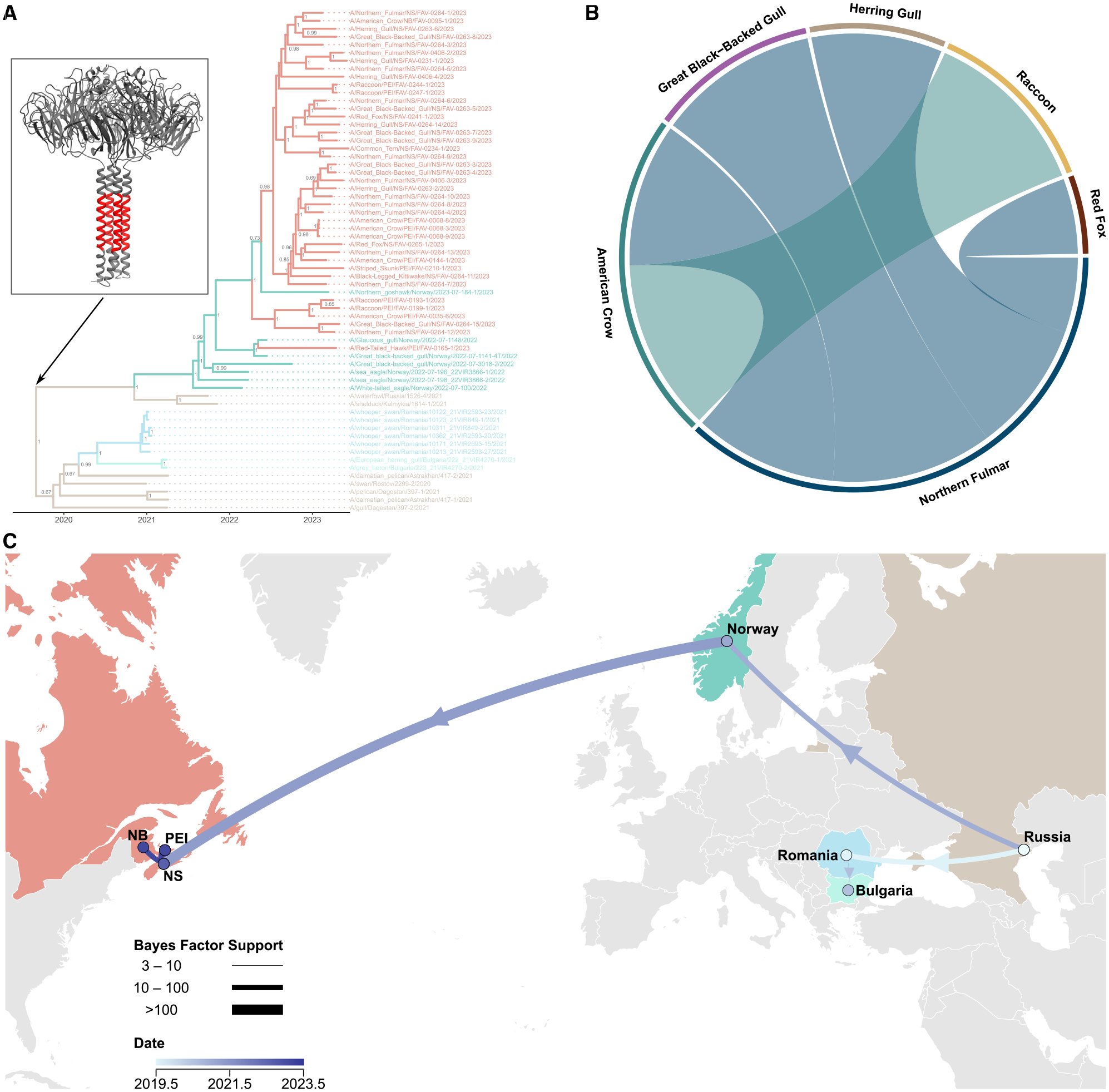
Bayesian analyses of viral WGS using BEAST to examine the relationship of A(H5N5) viruses (A) Bayesian timescaled maximum clade credibility tree. Inset contains a tetrameric model of NA with the region containing the 22-amino acid stalk deletion highlighted in red; PDB: 6CRD. (B) Chord diagram showing Bayesian host transition between species in Canadian A(H5N5) isolates; internal coloring matching the outer ring indicates the source species. Outer rings not matching internal coloring indicate sink species. Chords are statistically supported (Bayes factor ≥3.0). (C) Bayesian phylogeographic reconstruction of A(H5N5) dissemination (2020–2023) between European countries and the introduction of the virus into North America.

BLAST and phylogenetic analyses were performed to probe the origin of the Canadian A(H5N5) viruses. The NA segment of all Canadian A(H5N5) viruses described in this study contained a deletion of 22 amino acids in the stalk region (A(H5N5)-Δstalk; Figure 2A).

Time-resolved and maximum-likelihood (ML) inferred using partitioned full-genome sequences and individual genome segments demonstrated that the A(H5N5) virus sequences from Norway and Atlantic Canada clustered together (Figures 2A and S1). Based on the GenoFLU nomenclature, all A(H5N5)-Δstalk viruses were classified as clade 2.3.4.4b genotype A6 with a fully EA genetic constellation (PB2:ea6, PB1:ea6, PA:ea6, HA:ea6, NP:ea6, NA:ea6, MP:ea6, NS:ea6), with no evidence of reassortment. In contrast, the clade 2.3.4.4b A(H5N1) viruses currently circulating in the Americas have undergone reassortment, acquiring a variety of North American (NAm) lineage gene segments (Figure S1).

In ML trees of individual segments, similar clustering patterns were observed. Figures 2A and S1 show that the earliest A(H5N5) detection in Canada (A/American_Crow/PEI/FAV-0035-6/2023) and earliest mammal detection in raccoons (A/Raccoon/PEI/FAV-0193-1/2023 and A/Raccoon/PEI/FAV-0199-1/2023) were similar viruses (time of most recent common ancestor [tMRCA] December 18, 2022; 95% highest posterior density (HPD): November 21, 2022–January 8, 2023), even though they were temporally separated by 3 months. This contrasted with the second detection in Canada only 3 weeks later (A/American_Crow/PEI/FAV-0068-3/2023, A/American_Crow/PEI/FAV-0068-8/2023, and A/American_Crow/PEI/FAV-00689/2023) that was not closely related to A/American_Crow/PEI/FAV-0035-6/2023, even though they were collected within 100 m of each other. The earliest Canadian and mammalian viruses clustered with A/Northern_Fulmar/NS/FAV-0264-12/2023 and A/Great_Black-Backed_Gull/NS/FAV-0264-15/2023, both from Sable Island, and had a tMRCA of July 19, 2022 (95% HPD: May 2, 2022–September 28, 2022). Together, these data suggest that A(H5N5) was introduced to the same geographic area twice, with Sable Island being the potential source. The lone sample from NB was from an American crow and clustered with A/Northern_Fulmar/NS/FAV-0264-1/2023, also from Sable Island. A(H5N5) sequences (A/Herring_Gull/NS/FAV-0263-2/2023, A/Great_Black-Backed_Gull/NS/FAV-0263-3/2023, and A/Great_Black-Backed_Gull/NS/FAV-0263-4/2023 versus A/Great_Black-Backed_Gull/NS/FAV-0263-5/2023) from Sable Island did not cluster together consistently, even when sampled on the same day (April 14, 2023). The phylogenetic clustering patterns observed indicated that Sable Island contained a pool of genetically diverse A(H5N5) viruses within seabirds that subsequently spread the virus to other Atlantic provinces.

Of note, A/Red-Tailed_Hawk/PEI/FAV-0165-1/2023 was genetically distinct from the other Canadian A(H5N5) viruses and was the only case detected in Charlottetown, PEI. It clustered with two Norwegian isolates from June 2022 (A/Glaucous_gull/Norway/2022-07-1148/2022 and A/Great_black-backed_gull/Norway/2022-07-1141-4T/2022), suggesting two separate yet likely simultaneous incursions of A(H5N5)-Δstalk viruses into Canada.

Almost all of the Canadian and Norwegian A(H5N5) viruses had HA-A156T (mature H5 numbering), a substitution that restores HA-N154 glycosylation. One virus from Canada and two viruses from Norway evolved an alternative substitution that restores glycosylation, HA-A156S (Table S2). Two separate substitutions independently gave rise to this phenotype. In contrast, HA-A156 S/T mutations that restore HA-N154 glycosylation are not seen in any Canadian clade 2.3.4.4b A(H5N1) isolates (2,118 isolates from GISAID).

The Bayesian time-resolved tree (Figure 2A) shows a tMRCA for the split between HA-A156 and HA-S/T156 on July 23, 2021 (95% HPD: April 26, 2021–October 16, 2021), while the tMRCA between HA-T156 and HA-S156 was November 1, 2021 (95% HPD: August 13, 2021–January 16, 2022) when using the whole-genome sequences (WGSs) partitioned by segment.

The A(H5N5)-Δstalk NA segments appeared similar to those from A(H12N5) viruses that circulated in Russia from 2017 to 2018 and Belgium in 2018 (Figure S1). An A(H5N8) virus appears to have reassorted with an A(HxN5) virus, possibly an A(H12N5) to generate an A(H5N5) virus. At some point, the A(H5N5) viruses acquired a 22-amino acid NA stalk deletion (positions 47–68 as compared to A/swan/Rostov/2299-2/2020, EA N5 numbering) before there was any spread detected beyond the Caspian Sea region (August 31, 2019; 95% HPD: January 30, 2019–March 11, 2020).

The H5 ML tree (Figure S1) shows that the A(H5N1) (November 2021) and A(H5N5) (January 2023) viruses that entered Canada were not closely related to each other.

### Bayesian analysis of host and geographic characteristics to infer migration dynamics

#### Local host dynamics

We examined Canadian host-transmission dynamics due to greater sequence and metadata availability over a short time period. Northern fulmars appeared to be a source of A(H5N5) virus, while herring gulls and GBBGs were sinks. Additionally, northern fulmars were likely sources of virus for both American crows and red foxes. Crows subsequently acted as the source of virus for raccoons (Figure 2B). Bayesian stochastic search variable selection (BSSVS) support for host transitions and phylogenetic topology suggest that the species from Sable Island were involved in the transmission of A(H5N5) viruses to mainland Canada.

#### Geographic spread

A(H5N5)-Δstalk viruses had the same genotype as A/swan/Rostov/2299-2/2020. Viruses of the same genotype were detected in wild swans in Romania in January 2021, in a heron and a gull in Bulgaria in March 2021, and again in Russia in both pelicans and gulls in April 2021. The earliest A(H5N5)-Δstalk virus was detected in Russian waterfowl in September 2021. The spread of A(H5N5)-Δstalk viruses was detected in a white-tailed eagle in Norway in February 2022, followed by the detection in American crows and raccoons from Canada in January and April 2023, respectively. Bayesian analysis supports the established time line and the transatlantic migration of A(H5N5) viruses from Norway to Canada (Figure 2C). The most support is seen for a NS-to-PEI transition. Again, Sable Island appeared to be the source of all Canadian viruses other than that detected in Charlottetown, PEI, which was the only Canadian isolate with an HA-A156S mutation. The analysis was repeated, with NS separated into mainland and Sable Island sampling locations. This, again, supported Sable Island as a pool of viral diversity and the source of A(H5N5) viruses in Atlantic Canada.

#### Antigenic analyses of A(H5N5) viruses

We next compared the antigenic properties of A(H5N5) A/Raccoon/PEI/FAV-0193-1/2023 and A/American_Crow/PEI/FAV-0035-6/2023 viruses to each other, to other H5 viruses, and to existing World Health Organization (WHO) candidate vaccine viruses (CVVs) by hemagglutination inhibition (HI) assay (Table 1). The two A(H5N5) viruses had similar antigenic profiles, as determined by reactivity with post-infection ferret antiserum to other H5 viruses. The A(H5N5) viruses reacted well with ferret antisera generated to the clade 2.3.4.4b WHO CVV CBER-RG8A (A/Astrakhan/3212/2020-like), but less well to antiserum generated to the representative North American CVV A/American Wigeon/SC/22-000345-001/2021 (H5N1).

#### Pathogenicity and transmission of A(H5N5) viruses in ferrets

**Figure 3.**
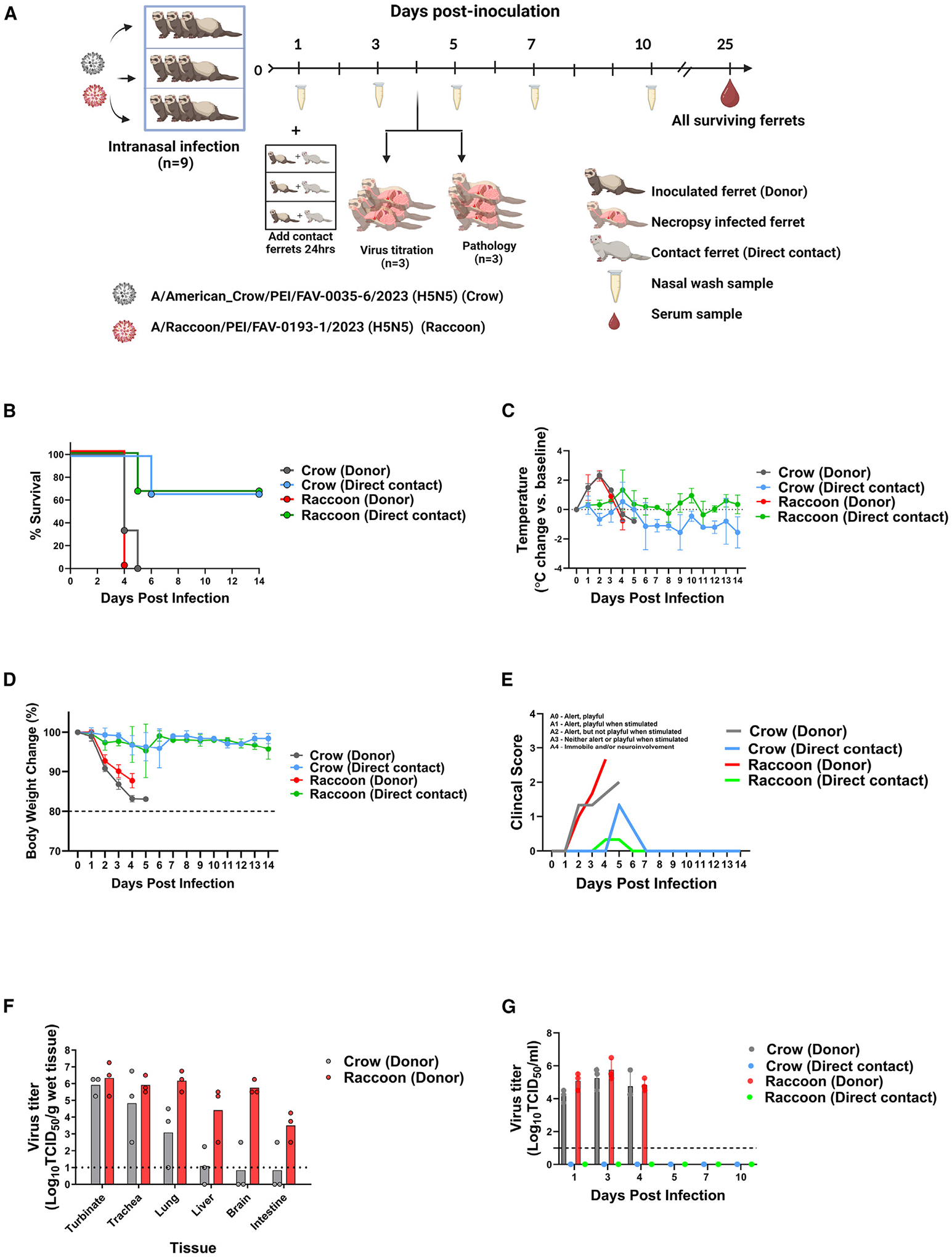
Pathogenicity and transmission potential of the A(H5N5) viruses in ferrets (A) Experimental design of ferret pathogenesis and transmission of A/American_Crow/PEI/FAV-0035-6/2023 (H5N5, Crow) and A/Raccoon/PEI/FAV-0193-1/2023 (H5N5, Raccoon) viruses. At 0 dpi, 9 ferrets were inoculated with 10^6^ median egg infectious doseunits of each A(H5N5) virus. Three inoculated ferrets (Donor) were individually co-housed with 3 naive contact ferrets (Direct contact) on 1 dpi. Six ferrets were euthanized at 4 dpi for viral titration in tissues and pathology (*n* = 3 each). All ferrets were monitored for clinical signs of infection until day 14. Nasal wash samples were collected from both infected and direct contact ferrets at the indicated time points for virus titration. Serum samples were collected from all surviving ferrets at 25 dpi for seroconversion assays. (B–E) Survival curve (B), (C) temperature change of inoculated ferrets (values are the average ± SE for each group), (D) weight changes (ferret weight values are the average ± SE for each group), and (E) clinical scores of inoculated ferrets (*n* = 3 per virus). (F) Infectious viral titers from collected tissues (*n* = 3 ferrets). (G) Infectious viral titers from nasal washes (mean virus titer ± SD). Symbols represent each individual animal’s titer. Dashed lines indicate the lower limit of virus titer detection (1.0 log_10_ TCID_50_/mL).

To assess the pathogenicity and transmission potential of the A(H5N5) viruses for mammals, we assayed two representative viruses (A/Raccoon/PEI/FAV-0193-1/2023 and A/American_Crow/PEI/FAV-0035-6/2023) in the ferret model (Figure 3A). Rapid onset of severe clinical signs (100% of inoculated animals met humane endpoints by 4–5 days post-infection [dpi]; Figure 3B), including elevated body temperature (Figure 3C) and loss of body weight, were observed in both groups of infected ferrets (Figure 3D). There was no significant difference in clinical scores between animals infected with either virus (Figure 3E) or differences in viral titers in nasal washes and tissues collected from the respiratory system (Figures 3F and 3G). Peak mean nasal wash titers were 5.75 and 5.25 log_10_ median tissue culture infectious dose (TCID_50_)/mL for A/Raccoon/PEI/FAV-0193-1/2023 and A/American_Crow/PEI/FAV-0035-6/2023, respectively, on day 3 (Figure 3G). All ferrets infected with A/Raccoon/PEI/FAV-0193-1/2023 showed systemic viral spread, with considerable infectious virus in extra-pulmonary tissue, including brain (5.75 log_10_ TCID_50_/mL), liver (4.41 log_10_ TCID_50_/mL), and intestine (3.5 log_10_ TCID_50_/mL; Figure 3F). In comparison, the ferrets infected with A/American_Crow/PEI/FAV-0035-6/2023 had less virus in extra-pulmonary tissues (Figure 3F). While we were unable to detect infectious virus from nasal washes of naive direct contact ferrets, one (of three) from each virus group met humane endpoints by day 5 post-cohousing (Figures 3B and 3G). These animals showed clinical signs, including severe diarrhea, neurologic signs, and weight loss. Additionally, one of two surviving ferrets from the A/Raccoon/PEI/FAV-0193-1/2023 group seroconverted (HI = 320).

**Figure 4.**
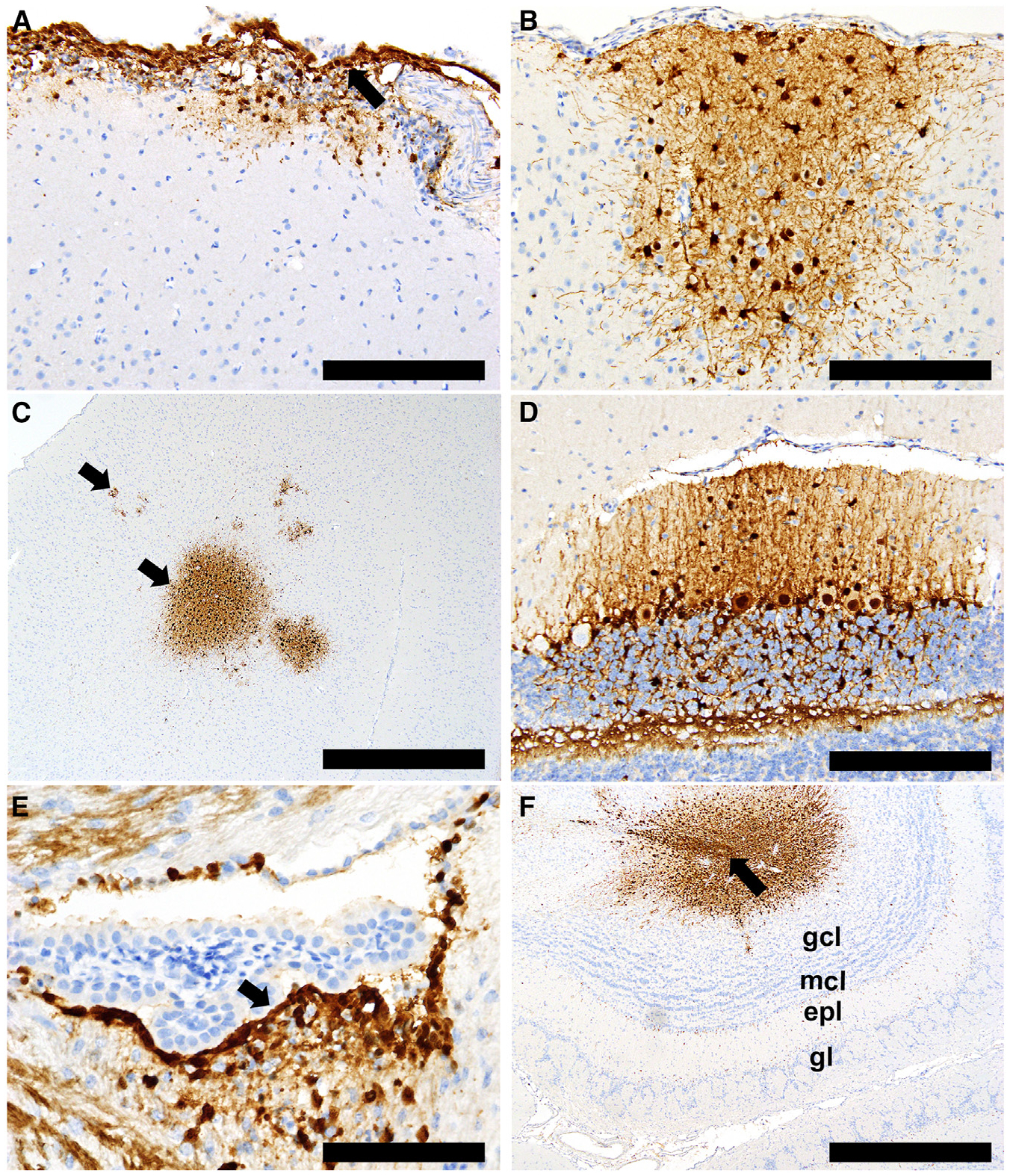
Ferrets infected with the A/Raccoon/PEI/FAV-0193-1/2023 (H5N5) virus (A–F) Histopathology and immunohistochemistry showing widespread CNS infection in several locations and cell types, including (A) meninges and submeningeal neuropil, (B) cortical astrocytes and neurons, (C) perivascular neuropil in cortex, (D) Purkinje cells and glial cells in cerebellum, (E) ventricular ependyma and subependymal cells (arrow) in midbrain, and (F) infection extending from the olfactory ventricle (arrow) into surrounding layear of the olfactory bulb. Granule cell layer (gcl); mitral cell layer (mcl); external plexiform layer (epl); glomerular layer (gl). Scale bars: (A), (B), and (D) 200 μm; (C) and (F) 1 mm; (E) 100 μm.

**Figure 5.**
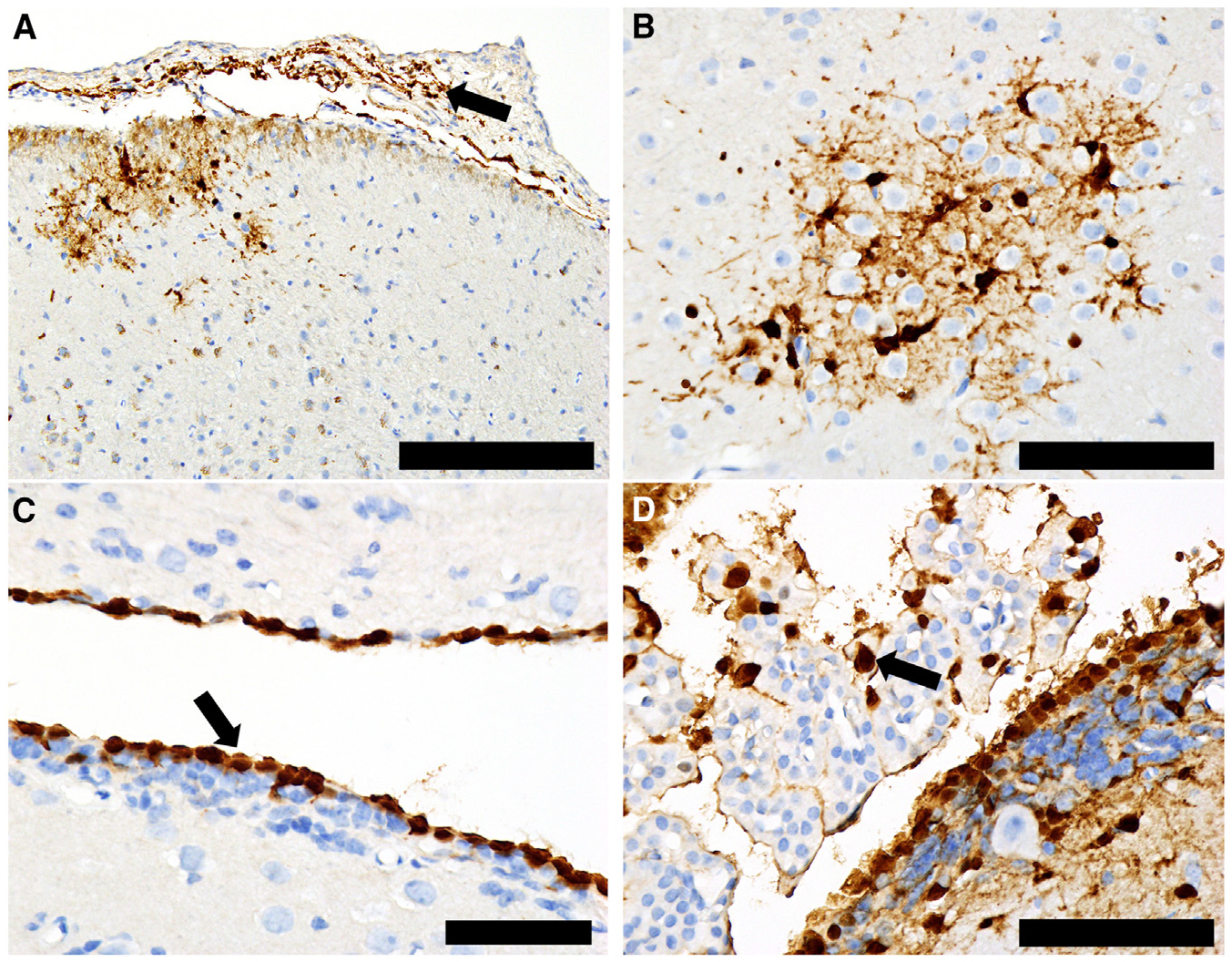
Ferrets infected with the A/American_Crow/PEI/FAV-0035-6/2023 virus (A–D) Histopathology and immunohistochemistry showing widespread CNS infection in several locations and cell types, including (A) meninges (arrow) and neuropil, (B) glial cells and some neurons in the cortex, (C) ependymal cells (arrow), and (D) choroid plexus (arrow) and periventricular ependyma/subependyma. Scale bars: (A) 200 μm; (B) and (D) 100 μm; (C) 50 μm.

Histopathology and immunohistochemistry findings from tissues collected from infected ferrets showed neuro-tropism of both viruses. Viral antigen was detected in different parts of the brain in cells, including neurons, microglia, ependyma, and cells within the meninges and choroid plexus. Viral antigen distribution in the CNS of A/Raccoon/PEI/FAV-0193-1/2023-infected ferrets suggested that multiple routes of neuroinvasion were involved, including direct spread from infected cells in the meninges (Figure 4A) to microglia and neurons, with secondary spread via neuronal axons (Figure 4B). The widely scattered foci of infection in other areas of the brain, including the cortex (Figure 4C) and cerebellum (Figure 4D), suggest hematogenous spread. Lastly, the extensive infection of ependymal cells (Figure 4E) with associated infection of subependymal neurons suggests CNS viral entry via the choroid plexus. Interestingly, viral infection of olfactory bulbs was detected in only one ferret (Figure 4F). Infection of the subependymal zone and diffuse infection of ependymal cells in the ventricular system indicate that infection likely originated in the lateral ventricle and spread to the subependymal zone via the rostral migratory stream. The overall distribution and cell tropism in the CNS of A/American_Crow/PEI/FAV-0035-6/2023-infected ferrets were essentially the same. There was evidence of local spread from infected meninges to adjacent neuropil (Figure 5A), widely scattered foci of infected microglia and neurons in other areas of the brain (Figure 5B), extensive infection of ependymal cells (Figure 5C), and scattered infected cells in the choroid plexus and subependymal zone located near ventricles (Figure 5D).

#### Antiviral susceptibility

The susceptibility of the A(H5N5) viruses to NA inhibitors (NAIs) and the cap-dependent endonuclease inhibitor (CENI) baloxavir was assessed by genotypic and phenotypic approaches. Analysis of all A(H5N5) virus sequences from 2021 through 2023 did not identify any of the established NAI resistance markers. All 41 viruses isolated in Canada in 2023 contained the NA-I117T substitution that has been previously shown to confer reduced inhibition (RI) to oseltamivir/zanamivir in HPAI A(H5N1) viruses.^49^ Substitutions in the polymerase acidic (PA) protein associated with RI by CENI (PA-E23 G/K, PA-K34R, PA-A36V, PA-A37T, PA-I38 M/T, and PA-E199G) were absent from the Canadian A(H5N5) viruses. One virus isolated from a swan in Sweden (A/Mute Swan/Sweden/SVA210303SZ0392/KN000806/Kal/2021) had the PA-E199G substitution, which may cause >3-fold reduced sensitivity to baloxavir.^50^ The Canadian viruses did have a PA-T40A substitution that is located close to the highly conserved isoleucine residue (I38) in the PA catalytic site. While this substitution is also prevalent among European A(H5N5) viruses (24.1%; 13/54), the frequencies are low among HPAI A(H5N1) (0.07%; 4/6,162), and seasonal H3N2 (0.02%; 9/38,741) viruses.

The *in vitro* susceptibility of A/Raccoon/PEI/FAV-0193-1/2023 and A/American_Crow/PEI/FAV-0035-6/2023 to the NAIs oseltamivir, zanamivir, and peramivir was determined by NA inhibition fluorometric assays using fluorogenic 2’-(4-methylumbelliferyl)-α-d-*N*-acetylneuraminic acid substrate.^51^ Plaque reduction assays and Influenza replication inhibition NA-based assays were used for measuring sensitivity to baloxavir.^52^ Phenotypic testing confirmed both viruses to be susceptible to antivirals with half-maximal inhibitory concentration/half-maximal effective concentration values being ≤2-fold higher than clade 2.3.4.4.b reference viruses (Table S3). As such, NA-I117T and PA-T40A do not confer RI to NAIs and CENI, respectively, in the context of A(H5N5) viruses.

#### Receptor binding properties

Both A(H5N5) isolates examined preferentially bound sialic acid receptors with a 3’SLN-linked sialic acid (preferred AIV receptor), rather than 6’SLN-linked sialic acid (preferred human influenza virus receptor) (Figure S2).

## DISCUSSION

In the present study, we document the incursion of GsGd-lineage A(H5N5) viruses into the Atlantic provinces of Canada, infecting wild birds, and subsequent spillover, causing fatal infections in numerous mammalian species.

### Transatlantic migration

**Figure 6.**
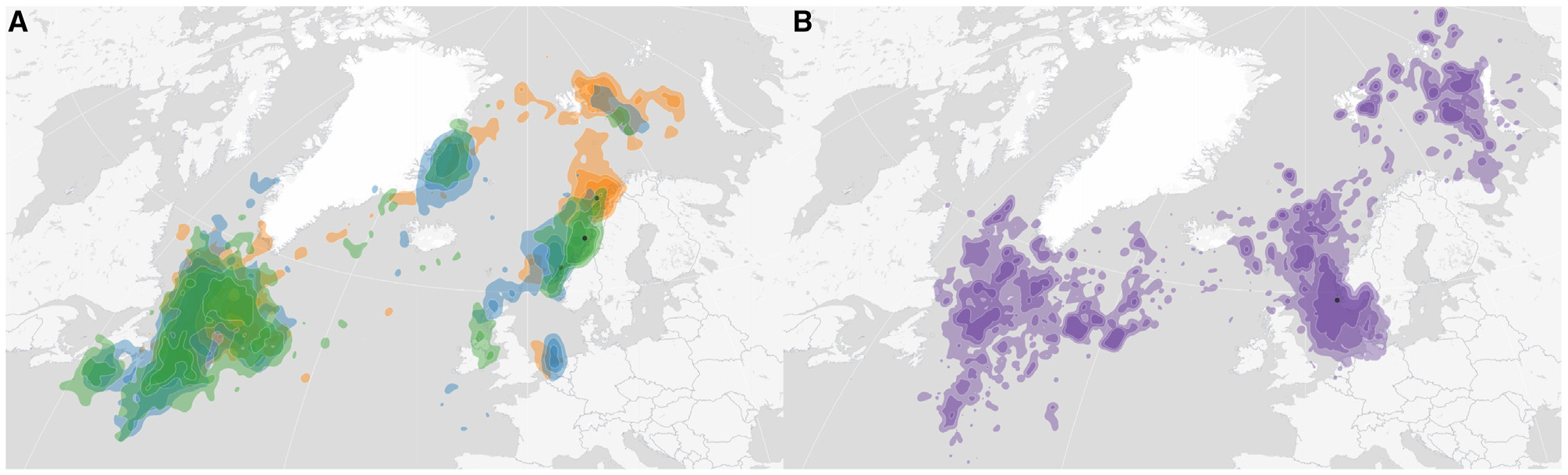
Migration pattern of black-legged kittiwakes and northern fulmar from Norwegian colonies (A) Black-legged kittiwake, 2021–2022; Anda, orange; Runde and Ålesund, blue; Sklinna, green. (B) Northern fulmar, 2011–2022; Jarsteinen, purple.

Millions of pelagic seabirds from different continents congregate in the North Atlantic year-round,^53^ suggesting a possible route for the spread of A(H5N5) viruses from Europe to North America.^32^ Mapping (Figure 6; Norwegian SEATRACK project http://www.seapop.no/en/seatrack/) transatlantic migration routes of northern fulmar and black-legged kittiwakes^54^ shows that Sable Island is within the range of pelagic seabirds from Norway. Earlier IAV sequences from gulls sampled in Newfoundland grouped with virus sequences from Norwegian gulls, suggesting virus transmission has previously occurred between these locales.^55^

GBBGs are one possible host for carrying A(H5N5) viruses from Europe to Canada,^32,56^ as they are known to travel across the Atlantic Ocean and are susceptible to IAV. Although a definitive role for these birds in the transatlantic spread of IAV is missing due to inadequate sampling depth,^57^ evidence is building for their role as spreaders. Four GBBGs infected with A(H5N5) virus were detected at the end of January and beginning of February 2023 in Cape Cod, Massachusetts, a peninsula that extends into the Atlantic Ocean.^56^ Further evidence comes from the fact that in both instances of clade 2.3.4.4b virus (A(H5N1) and A(H5N5)) spread to North America, the earliest detections in migratory birds were in GBBGs (November 2021, H5N1; January 2023, H5N5).

Sable Island also appears to be a source of A(H5N5) virus, acting as the likely origin of Canadian spread (Figure 2) after the transatlantic migration of pelagic seabirds. Of note, while Sable Island is home to the largest colony of gray seals in the world,^58^ a host known to be susceptible to IAV,^59^ no reports of H5 infection have been made in these animals, unlike the situation reported elsewhere.^31^

While Iceland could be geographically implicated in the Eurasia to North America spread of A(H5N5) viruses, the virus was not reported there until September 2023^60^ (this detection was followed by others in the United Kingdom^61^). Similarly, A(H5N5) viruses were detected in a northern fulmar and glaucous gull in September and October, respectively, in Disko Bay, Greenland.^60^ These detections suggest that the spread of IAV can also be mediated via shorebirds,^32^ although more intensive sampling is needed to support this assertion.

Clade 2.3.4.4b A(H5N5) viruses had caused several previous outbreaks in Europe (2016–2017, 2020–2021).^62^ In the 2016–2017 European outbreak, the viruses were detected in Kamchatka, Russia,^63^ followed by the Netherlands and Germany.^64^ Possibly due to the timing of the outbreak relative to bird migration, these viruses did not spread further.^64^ A(H5N5) viruses were next detected in Germany and Denmark during the 2020–2021 outbreak. While the 2020–2021 A(H5N5) viruses spread across Europe,^62,65^ they did not reach North America^66^ (Table S4). Of note, the 2021 A(H5N1) viruses were detected in the Netherlands in months similar to those in the 2022 A(H5N5) viruses in Norway, being early in the year (Table S3). In both instances, early virus detection in Europe led to late detections of similar viruses in Canada. In the 2016 and 2020 European A(H5N5) outbreaks, which did not spread to Canada, virus detections were made late in the season, which is supportive of the importance of outbreak timing in risk of intercontinental spread.

### Mutations in NA and HA

A characteristic feature of the 2023 A(H5N5) viruses was the presence of the same 22-amino acid deletion in the NA stalk as described in earlier studies.^41^ Only one instance of a stalk deletion in N5 subtype viruses has been described.^67^ There is no overlap with the contemporary N5 deletion. Such deletions in other IAV NA subtypes are typically associated with adaptation to gallinaceous hosts,^67,68^ and HPAI A(H5N1) viruses with NA deletions display higher pathogenicity in chickens, ducks, and mice.^69–72^ According to previous studies, viruses with partial stalk truncations have increased thermal and low-pH stability, yet reduced NA activity.^71,73^ NA truncation and NA deglycosylation reduce virus transmissibility in ferrets and mice,^74,75^ but promote the systemic spread of HPAI viruses, increasing pathogenicity.^75^ It is unclear what role the deletion has played in replication of the A(H5N5) viruses in shorebirds and gulls or in their intercontinental spread.

The HA-T156A (mature H5 numbering) substitution, present in some 2.3.4.4 A(H5) viruses, has been linked to dual receptor binding properties.^76^ HA-T156A destroys the glycosylation sequon Asn-X-Ser/Thr,^77^ impacting immunogenicity,^78^ antigenicity,^79,80^ and host immune response escape.^81^ The HA-T156A mutation has also been associated with the emergence of clade 2.3.4.4 viruses that are paired with NA segments other than N1.^82,83^ As the A(H5N5)-Δstalk virus spread from Norway to Canada, it acquired the reverse HA-A156T mutation, restoring the glycosylation motif. Almost all global 2023 A(H5N5) isolates (41 Canadian and 1 Norwegian) contained this mutation (Table S1). One exception is the A(H5N5)-Δstalk virus, isolated from the Canadian red hawk sample (A/Red-Tailed_Hawk/PEI/FAV-0165-1/2023), which has an HA-A156S mutation, also restoring the N-glycosylation sequon. This hawk sample shared similarity with two Norwegian isolates from 2022 (A/Glaucous_gull/Norway/2022-07-1148/2022 and A/Great_black-backed_gull/Norway/2022-07-1141-4T/2022; Figure 2A). When ancestral sequence reconstruction and BSSVS (Table S2) were combined with phylogenetic topology (Figure 2A), both HA-A156 S/T mutations appeared to have occurred independently, indicating two separate incursions of A(H5N5) viruses into Canada from Norway. This HA-A156 S/T mutation is not seen in any clade 2.3.4.4b A(H5N1) viruses currently circulating in Canada (2,118 isolates).

Recently, A(H5N1) isolates from Nigeria with an N1 stalk deletion^84^ and HA-A156S substitution were detected. In Europe, 1,956 A(H5Nx) viruses from 2020 to 2022 have been characterized.^85^ The majority of these viruses (1,939) harbored HA-A156, while only 7 viruses contained an NA stalk deletion. This contrasts reports from earlier years where the majority of NAs from A(H5N1) contained stalk deletions.^69,71,73^ This change might be associated with the proliferation and dominance of clade 2.3.4.4 viruses with HA-T156A substitutions. As balanced HA-NA activity is critical for IAV^86^ replication, it is tempting to speculate that the HA-A156T mutation balances the N5 stalk deletion. Increased HA glycosylation has been shown to lead to more viral shedding in the poultry respiratory tract.^71^ While HA-A156 can bind α−2,6-linked glycans, resulting in increased virulence,^87^ the HA-A156T reversion could allow avian species to shed virus more readily, and still maintain virulence. Supportive of this, we could show that the Canadian A(H5N5) viruses bound 3’SLN-linked sialic acid but not 6’SLN-linked sialic acid. While reassortants were detected in the 2016–2017 and 2020–2021 European A(H5N5) outbreaks,^24,64–66^ the A(H5N5)-Δstalk descendants do not appear to have reassorted over the past 2 years.^88^

### Risk for mammals

While infections with IAV containing N5 have been described in seals (H4N5)^89^ and swine (H10N5),^90^ to our knowledge there have been no previously reported cases of A(H5N5) infection in mammals. In the present study, we have 7 confirmed cases of mammalian A(H5N5) infections leading to deaths (4 raccoons, 2 red foxes, 1 striped skunk). While it is concerning that individual mammalian-adaptive mutations were detected in these animals, there was no evidence of mammal-to-mammal transmission. The high proportion of Canadian A(H5N5) detections in mammals (7 of the 41, 17%, total detections) is surprising. In contrast, of the 2,118 Canadian A(H5N1) detections, only 96 were from mammals (4.5%).

Ferrets have been the benchmark model for assessing risk to humans. In our hands, the A(H5N5)-Δstalk viruses were highly virulent in ferrets (100% mortality; Figure 3B) and transmitted, albeit inefficiently, to direct contact animals (as measured by clinical signs and seroconversion). Inefficient viral detection in nasal washes of directly contacted ferrets, together with observed clinical signs, including severe diarrhea, neurologic signs, and weight loss, suggest that infection may have initiated in the lower respiratory tract through small infectious droplets or extrapulmonary sites like the digestive system or nervous system, although tissue was not available to confirm this. A total of three amino acid substitutions were found in A/Raccoon/PEI/FAV-0193-1/2023 compared to A/American_Crow/PEI/FAV-0035-6/2023: PB2 (T271A), PB1 (N328K), and HA (I200V). These three mutations have previously been characterized with mammalian adaptation of IAVs and are possibly linked to the more robust systemic replication of A/Raccoon/PEI/FAV-0193-1/2023 in ferrets. Transmission of the A(H5N5)-Δstalk viruses is consistent with previous reports linking HA-T156A to airborne transmission among ferrets^87,91^ and guinea pigs.^92^ Similar ferret studies with the EA A(H5N1) viruses A/Fancy Chicken/Newfoundland/FAV-0033/2021 and A/American Wigeon/South Carolina/22-000345-001/2021 led to no mortality or transmission in infected ferrets.^93^

The A(H5N5)-Δstalk viruses appear to be neurotropic in mammals, as seen from the histologic lesions and immunostaining in ferret brain tissues (Figures 4 and 5). Neuroinvasion is known to occur in mammalian A(H5Nx) infection,^94^ and our ferret data are consistent with other cases of Canadian mesocarnivores from which clade 2.3.4.4b A(H5N1) viruses were isolated from brain samples.^55^ While the A(H5N5)-Δstalk viruses exhibit high mortality in the ferret model, they remained susceptible to antivirals such as NAIs and CENI (Table S3). Although the NA subtypes differ, results are consistent with clade 2.3.4.4b A(H5N1) resistance data.^95^

Although influenza viruses are well studied, many gaps in our knowledge remain. As IAV represents a global risk, intensive surveillance is required, especially in bird species found in migratory stopovers, where mingling of birds from different flyways occurs. Our data implicate pelagic seabirds in the transatlantic and local Canadian spread of A(H5N5) viruses that have maintained an NA stalk deletion. These viruses have been detected in wild mammals, where markers for mammalian adaptation (PB2-E627K) have been detected. Ferret pathogenesis studies with the EA genotype A(H5N5) viruses demonstrated their high virulence, which is in contrast to the early North American A(H5N1) viruses that required North American segments to induce mortality. Thus, while A(H5N5) viruses are comparably uncommon, their high virulence and mortality potential demand global surveillance and further studies to untangle the molecular markers influencing virulence, transmission, adaptability, and host susceptibility.

### Limitations of the study

In this study, we examined the genetic relationship between A(H5N5) viruses and the transmission capability of the earliest detected Canadian avian and mammalian A(H5N5) viruses in a ferret model. A limitation of this study is that no *in vitro* experiments were performed to examine the enzymatic activity and growth kinetics comparing truncated NA stalk and full-length stalk A(H5N5) viruses. Clade 2.3.4.4b A(H5N1) virus reassorted with NAm lineage IAVs shortly after its incursion. However, the clade 2.3.4.4b A(H5N5) has been circulating in Canada for over a year and has not reassorted with local NAm lineage IAVs. We have not confirmed if the stalk deletion and HA glycosylation led to a stable virus that is less likely to reassort.

## STAR★METHODS

### RESOURCE AVAILABILITY

#### Lead contact

Further information and requests for resources and reagents should be directed to and will be fulfilled by the lead contact, Yohannes Berhane (Yohannes.Berhane@inspection.gc.ca).

#### Materials availability

The full genome sequences of the 41 A(H5N5) cases from Canada generated in this study have been deposited at GISAID and are publicly available as of the date of publication under the Isolate IDs: EPI_ISL_18702952 to EPI_ISL_18702992. This study did not generate new unique reagents.

#### Data and code availability

This paper does not report original code. This paper analyzes existing, publicly available data. These accession numbers for the datasets are listed in the key resources table. Any additional information required to reanalyze the data reported in this paper is available from the lead contact upon request.

### EXPERIMENTAL MODEL AND SUBJECT PARTICIPANT DETAILS

Male Ferrets (Triple F Farms, Sayre, PA, USA). Procedures used in this study were reviewed and approved by the St. Jude Children’s Research Hospital Institutional Animal Care and Use Committee (IACUC, Protocol number 428). Madin–Darby canine kidney (MDCK) cells (ATCC) were used.

### METHOD DETAILS

#### Clinical samples from animals

Samples, including swabs and tissues were collected between January 9^th^, 2023 and June 9^th^, 2023 from animals found dead or euthanized at various rehabilitation centers or at Canadian Wildlife Health Cooperative regional center wildlife clinics in the Atlantic provinces of Canada. Species of animals and their geographic locations are depicted in Figure 1B. Clinical samples collected from animals were tested at regional Animal Health Laboratories or provincial Canadian Animal Health Network (CAHSN) laboratories. All IAV matrix-positive samples were submitted to the National Centre for Foreign Animal Disease laboratory in Winnipeg for confirmatory testing.

#### RNA extraction and virus detection

Total RNA was extracted from clinical samples (swabs and tissues) and virus isolates using the MagMAX-96 Viral RNA Isolation Kit with the KingFisher Duo Prime platform (ThermoFisher Scientific, Waltham, MA, USA). The presence of IAV genomic material was verified using the matrix gene-specific qRT–PCR, followed by H5-specific qRT–PCR.^96,97^

#### Virus isolation and titration

For virus isolation, IAV PCR positive samples from submissions were propagated through the allantoic cavities of 9 to 11-day-old embryonated specific pathogen-free (SPF) chicken eggs. Viral titers for the A/American_Crow/PEI/FAV-0035-6/2023 (H5N5) and A/Raccoon/PEI/FAV-0193-1/2023 (H5N5) viruses were determined by the 50% egg infectious dose (EID_50_).

#### Viral sequencing and assembly

Amplicon samples were subject to library preparation using the Oxford Nanopore Rapid Barcoding Kit (SQK-RBK110.96) and sequenced using MinION R9.4.1 Flow Cells (FLO-MIN106D) on an Oxford Nanopore GridION sequencer (Oxford Nanopore Technologies).^94^ The raw Nanopore signal data was basecalled and demultiplexed with Guppy (v6.3.9) using the super-accurate basecalling model. Raw sequence data was processed using nf-flu (v3.3.5; https://github.com/CFIA-NCFAD/nf-flu) and Geneious Prime 2023.0.4 with Minimap2 (v2.2.0).^98^

#### Sequence collection

All A(H5N5) sequences, other than those generated for this study, were retrieved from the National Center for Biotechnology Information (NCBI) Influenza Virus Sequence Database and the Global Initiative on Sharing All Influenza Data (GISAID) EpiFlu database (https://gisaid.org)^99^ from 2015-01-01 until 2023-08-31. Reference sequences of A(H5N1) viruses from GenoFLU^100^ and the top 25 non-A(H5N5) hits to A/American_Crow/PEI/FAV-0035-6/2023 (H5N5) virus from a Basic Local Alignment Search Tool (BLAST) search on both GISAID and NCBI were included in phylogenetic analysis. Sequences were aligned using MAFFT v7.490^101^ and trimmed to contain major open reading frames except for M and NS segments, which were trimmed to 982 and 838 nucleotides, respectively. Sequences were manually cleaned and those containing at least 90% of the ORF were screened for recombinants with RDP5 (v5.46)^102^ using default settings with linear sequences, and results were retained for further phylogenetic analysis.

#### Maximum-likelihood phylogenetics

Maximum-likelihood (ML) trees and best-fitting nucleotide substitution models (using ModelFinder^103^) were inferred using IQ-TREE v2.2.2.7^104^ and 1000 replicates were used for the Shimodaira–Hasegawa approximate likelihood ratio test. TreeTime (v0.11.1)^105^ was used to reconstruct the most likely ancestral sequence for HA. Genome constellations for contemporary A(H5N5) viruses are named for consistency with GenoFLU.^100^ All resultant phylogenetic trees were visualized using ggtree.^106^

#### Bayesian phylogenetics

A(H5N5) viruses similar to A/swan/Rostov/2299-2/2020 and possessing eight segments were examined and reassortants were removed (*n* = 8; no NA stalk deletion) resulting in 62 whole-genome sequences (WGS). These sequences were partitioned by segment and used to estimate the timing and likelihood of transmissions between geographic locations and hosts by phylogeographic diffusion in discrete space using BEAST v1.10.4.^107,108^ The partitioned WGS alignments were used to estimate a time-scaled phylogenetic tree under the SRD06 model of nucleotide substitution (HKY_112_ + CP_112_ + г4_112_) except for M and NS, which used a GTR nucleotide substitution model, a relaxed molecular clock with lognormal distribution and an exponential population coalescent tree prior. Sampling location [the Canadian provinces of New Brunswick (NB), Nova Scotia (NS), and Prince Edward Island (PEI), and the countries Norway, Bulgaria, Romania and Russia], viral host (Northern fulmar, American crow, gray heron, eagle, swan, shel-duck, herring gull, pelican, red-tailed hawk, gull, great black-backed gull, red fox, raccoon, Northern goshawk, striped skunk, black-legged kittiwake and common tern), and the residue at HA-156 (mature H5 numbering; alanine or A, serine or S, threonine or T) were attached to each tree tip as discrete character states. Ancestral character states for location, host and HA-156 residue were reconstructed by the asymmetric substitution model and social networks were inferred with the Bayesian stochastic search variable selection (BSSVS) procedure. Two independent Markov Chain Monte Carlo chains (400,000,000 steps, sampled every 10,000) were run. The first 10% of samples from each chain were burned and assessed for convergence (effective sample size >200) using Tracer v1.7.2.^110^ Post burn-in samples from independent chains were combined using LogCombiner v1.10.4 and a maximum clade credibility (MCC) tree was produced using TreeAnnotator v1.10.4.^108^ The posterior distribution of indicator values from the BSSVS procedure was used to conduct Bayes factor (BF) tests to garner statistical support for location, host, and HA-156 residue transitions using SpreaD3 (v0.9.7.1).^111^ Transitions with BF < 3.0 and/or posterior probabilities <0.60 were discarded from the dataset, along with non-Canadian host transition species. The MCC tree was used to plot location state transitions on a world map with SpreaD3. The width of each transition line was manually adjusted to represent the underlying BF support (substantial support: 3.0 ≤ BF < 10.0, very strong support: 10.0 ≤ BF < 100.0, and decisive support: BF ≥ 100.0).^107,112^ Relative median host transition rates were calculated using Tracer v1.7.2 and visualized using the circlize (v0.4.15) package^113^ in R v4.3.2.

#### Hemagglutination inhibition (HI)

A(H5N5) isolates were analyzed by a hemagglutination inhibition (HI) assay against reference ferret antisera raised against CBER-RG8A (A/Astrakhan/3212/2020-like), A/American wigeon/SC/22-000345-001/2021, and A/Bald eagle/FL/W22-134-OP/2022 viruses. HI assays were performed with 0.5% chicken red blood cells.

#### Pathogenesis and transmission in ferrets

Six-month-old male ferrets (*n* = 9; Triple F Farms, Sayre, PA, USA) were infected intranasally with 10^6^ EID_50_ of A(H5N5) viruses diluted in 500 μL of phosphate-buffered saline (PBS). Virus inoculation of ferrets was performed under light anesthesia of inhalant isoflurane vaporized in O_2_. After one day post-infection (dpi), three naive ferrets were placed in the same cages with infected ferrets as the contact recipient ferrets. Ferrets were monitored daily for any clinical signs including body temperature changes, body weight loss, respiratory disorders, stool consistency, and neuropathologic signs. Nasal washes were collected from all surviving ferrets at 1, 3, 5, 7, and 10 dpi and ketamine was used to induce sneezing. At 4 dpi ferrets (*n* = 3) were euthanized, and tissue samples (nasal turbinates, trachea, lung, brain, liver, and intestine) were collected for virus titration in MDCK cells (American Type Culture Collection, Manassas, VA, USA) using median tissue culture infectious dose (TCID_50_) assays. Animals reaching the humane endpoint were euthanized. At 25 dpi, sera was collected from all surviving ferrets for seroconversion an using HI assay. Another 3 infected ferrets from each group were euthanized and fixed tissues were subjected to histopathology and immunohistochemistry (IHC) - see Immunohistochemistry section.

#### Ethics approval

Animal experiments were approved by the St. Jude Children’s Research Hospital Institutional Animal Care and Use Committee (IACUC, Protocol number 428).

#### Immunohistochemistry

IHC was performed using the Ventana Discovery Ultra Autostainer (Roche Ventana, Tucson, Arizona, USA) following the manufacturer’s instructions. Five μm formalin-fixed and paraffin-embedded sections were initially heated for 4 min at 72°C and placed in EZ prep solution (#950–102, Roche Ventana) for deparaffinization. Antigen retrieval was performed at 95°C in Cell Conditioning Solution 1 (CC1, #950–124, Roche Ventana) for 56 min. A polyclonal primary goat polyclonal antibody (US Biological, Swampscott, MA) against influenza A, USSR (H1N1) at 1:1000 and a secondary biotinylated donkey anti-goat antibody (US Biological) at 1:200 were used on tissue sections. The DISCOVERY ChromoMap DAB detection kit (#760–159, Roche Ventana) was used as detection system. Tissue counterstaining was performed with Hematoxylin II solution (#790–2208, Roche Ventana).

#### NA-inhibitor susceptibility assays

##### Enzymatic inhibition assays

A fluorescence-based NA inhibition assay was conducted.^51^ The NA activity of each virus was standardized to relative fluorescent unit equivalents of 10 μM 4-methylumbelliferone (4-MU).^114^ Fluorescent NA-cleaved MUNANA (2’–[4-methylumbelliferyl]-α-D-N-acetylneuraminic acid) substrate was measured with a range of NA inhibitors or NAI (oseltamivir carboxylate [oseltamivir], zanamivir and peramivir) concentrations. The half-maximal inhibitory concentrations (IC_50_) were determined in GraphPad Prism 8.4.3 software from the sigmoidal dose-response (variable slope) equation. IC_50_-fold changes were calculated relative to the reference virus of the respective subtype/clade/lineage. The NAI susceptibility phenotypes were defined according to the World Health Organization Antiviral Working Group criteria: normal inhibition (NI), <10-fold; reduced inhibition (RI), 10- to 100-fold; highly reduced inhibition (HRI), >100-fold.

##### Replication inhibition assays

Influenza Replication Inhibition NA-based Assay (IRINA) was implemented according the Centers for Disease Control and Prevention protocol.^52^ Virus inoculum was normalized to 1.9 nM/well of 4-MU and incubated for 24 h with the cap-dependent endonuclease inhibitor (CENI), baloxavir, in MDCK cells without TPCK-treated trypsin to achieve a single-cycle virus replication. NA activity from the infected cells was measured and half-maximal effective concentrations (EC_50_) were calculated as described for NA Inhibition. EC_50_-fold changes were derived by comparison to the reference viruses without potential CENI RI-associated substitutions. An arbitrary threshold of a ≥3-fold increase in median EC_50_ was used as RI phenotype.^115^

##### Endonuclease inhibitor susceptibility

MDCK cells at confluence (1×10^6^ cells/well) were inoculated with influenza viruses, yielding 50 to 100 plaque-forming units (PFUs) per well. After a 1 h incubation at 37°C, the monolayer was overlaid with 0.45% immunodiffusion-grade agarose (MP Biomedical) in DMEM supplemented with 4% bovine serum albumin, 1 μg/mL TPCK-treated trypsin, and baloxavir at a range of concentrations (1 p.m.−10 μM). At 60 to 72 hpi, the cells were stained with 0.7% crystal violet in 10% formaldehyde. PFU/well were enumerated, and EC_50_s were determined using the log (inhibitor) versus response logistic nonlinear regression.

##### Receptor binding assay

Fetuin-coated 96-well plates were washed using washing buffer (0.23X PBS with 0.01% Tween 80) then blocking solution (1X PBS containing 1% BSA) was added to all coated wells. 100μL of wash virus containing 64 HA units of each virus was added and all plates were incubated overnight at 4 °C. Biotinylated sialylglycopolymers, 3’SLN-C3-PAA-biot (3’SLN) or 6’SLN-C3-PAA-biot (6’SLN) (GlycoNZ), were serially diluted from 10 to 0.156 μg/well in reaction buffer (1X PBS with 0.02% Tween 80, 0.02% BSA, and 5 μM Zanamivir) then added to the washed plates containing bound virus and incubated overnight at 4 °C. Plates were washed 4 times then horseradish peroxidase-conjugated streptavidin (1:2000) was added to the plates and incubated for 1 h. Substrate was added to the plates and kept for 10 min at room temperature followed by the addition of 1 N H_2_SO_4_ to stop the reaction.

### QUANTIFICATION AND STATISTICAL ANALYSIS

Data were analyzed using two-way ANOVA with Tukey’s multiple-comparison post hoc test, and univariant log rank analysis (survival curves) in GraphPad Prism v10.

### ADDITIONAL RESOURCES

Norwegian SEATRACK project: http://www.seapop.no/en/seatrack/

## Supplementary Material

1

2

3

## Figures and Tables

**Table 1.** HI assay of early 2023 clade 2.3.4.4b A(H5N5) viruses using 0.5% chicken RBCs

Antigen	Subtype	Clade	A/Astrakhan/3212/2020-like	A/American Wigeon/South Carolina/22-000345-001/21	A/Bald Eagle/Florida/W22-134-OP/2022	Collection date	Passage history
CBER-RG8A (A/Astrakhan/3212/2020-like)	H5N8	2.3.4.4b	320^a^	40	160		V1E2/E1
A/American Wigeon/South Carolina/22-000345-001/21	H5N1	2.3.4.4b	160	80^a^	160		E2/E1
A/Bald Eagle/Florida/W22-134-OP/2022	H5N1	2.3.4.4b	80	40	80^a^		E2
A/Raccoon/PEI/FAV-0193-1/2023	H5N5	2.3.4.4b	80	10	10	May 2023	E1/E1
A/American_Crow/PEI/FAV-0035-6/2023	H5N5	2.3.4.4b	80	10	10	February 2023	E1/E1

a The titers of serum samples with homologous viruses.

**Table:** KEY RESOURCES TABLE

REAGENT or RESOURCE	SOURCE	IDENTIFIER
Antibodies		
Anti-influenza A virus (A/USSR/1977 (H1N1) primary goat polyclonal	US Biological Life Sciences	I7650-05E
Donkey anti-goat biotinylated secondary	US Biological Life Sciences	I1904-28B
CBER-RG8A	In house	N/A
A/American wigeon/South Carolina/22-000345-001/21	In house	N/A
A/bald eagle/Florida/W22-134-OP/2022	In house	N/A
Bacterial and virus strains		
A/American_Crow/PEI/FAV-0035-6/2023 (H5N5)	This study	N/A
A/Raccoon/PEI/FAV-0193-1/2023 (H5N5)	This study	N/A
Influenza A/CA/04/2009 (H1N1)	CDC	
Biological samples		
Tissue and swab samples	This study	N/A
Chemicals, peptides, and recombinant proteins		
Chicken Red Blood Cells	Rockland Immuno.	R202-0050
EZ prep solution	Roche Ventana	950–102
Cell Conditioning Solution 1	Roche Ventana	950–124
DISCOVERY ChromoMap DAB detection kit	Roche Ventana	760–159
Hematoxylin II solution	Roche Ventana	790–2208
4-methylumbelliferone (4-MU)	Sigma	M1508
2–[4-methylumbelliferyl]-α-D-N-acetylneuraminic acid	Sigma	M8639
Oseltamivir carboxylate	Medchem Express	HY-13318
Zanamivir	Medchem Express	HY-13210
Peramivir	Medchem Express	HY-17015A
Baloxavir acid	Medchem Express	HY-109025A
TPCK-treated trypsin	Fisher Scientific	PI20233
3′SLN-C3-PAA-biot	GlycoNZ	0036-BP
6′SLN-C3-PAA-biot	GlycoNZ	0997-BP
Critical commercial assays		
MagMAX-96 Viral RNA Isolation Kit	ThermoFisher Scientific	AMB18365
Rapid Barcoding Kit 96	Oxford Nanopore Technologies	SQK-RBK110.96
MinION R9.4.1 Flow Cells	Oxford Nanopore Technologies	FLO-MIN106D
Deposited data		
A(H5N5)-Δstalk virus consensus sequences	This paper	GISAID: EPI_ISL_18702952 to 18702992
Influenza A sequences	GenBank	See Table S5
Influenza A sequences	GISAID	See Table S6
Experimental models: Cell lines		
MDCK cells	ATCC	CCL-34; RRID:CVCL_0422
Experimental models: Organisms/strains		
Ferrets - male	Triple F Farms	RRID:NCBITaxon_9669
Oligonucleotides		
Primer: Inf-A-2009 F: AGATGAGTCYTCTAACCGAGGTCG	Weingartl et al.^96^	N/A
Primer: Inf-A-2009 R: TGCAAARACAYYTTCMAGTCTCTG	Weingartl et al.^96^	N/A
Probe: M +64 P: FAM-TCAGGCCCCCTCAAAGCC GA-BHQ1	Weingartl et al.^96^	N/A
Primer: H5 Protected by supplier MTA	Modified Spackman et al.^97^	N/A
Primer: H5 Protected by supplier MTA	Modified Spackman et al.^97^	N/A
Probe: H5 Protected by supplier MTA	Modified Spackman et al.^97^	N/A
Software and algorithms		
Guppy v6.3.9	Oxford Nanopore Technologies	https://community.nanoporetech.com/downloads
nf-flu v3.3.5	CFIA-NCFAD	https://github.com/CFIA-NCFAD/nf-flu
Geneious Prime 2023.0.4	GraphPad Software Inc.	https://www.geneious.com/
Minimap2 v2.2.0	Li^98^	https://www.geneious.com/plugins/minimap2/
MAFFT v7.490	Katoh and Standley^99^	https://www.geneious.com/plugins/mafft-plugin/
RDP5 v5.46	Martin etal.^100^	http://web.cbio.uct.ac.za/~darren/rdp.html
GenoFLU	Youk et al.^101^	https://github.com/USDA-VS/GenoFLU
IQ-TREE v2.2.2.7 (ModelFinder)	Nguyen et al.^102^; Kalyaanamoorthy et al.^103^	https://github.com/iqtree/iqtree2
TreeTime v0.11.1	Sagulenko et al.^104^	https://github.com/neherlab/treetime
BEAST (LogCombiner, TreeAnnotator) v1.10.4	Suchard et al.^105^	https://beast.community/
Tracer v1.7.2	Rambaut et al.^106^	https://github.com/beast-dev/tracer/
SpreaD3 v0.9.7.1	Bielejec et al.^107^	https://rega.kuleuven.be/cev/ecv/software/SpreaD3
R	The R Foundation	https://www.r-project.org/
ggtree	Yu et al.^108^	https://bioconductor.org/packages/ggtree/
circlize v0.4.15	Guetal.^109^	https://github.com/jokergoo/circlize
GraphPad Prism v8.4.3	GraphPad Software Inc.	https://graphpad.com

## Highlights

- Seabirds likely enabled multiple incursions of A(H5N5) virus from Europe to Canada
- Some A(H5N5) viruses possessed the mammalian adaptation marker PB2-E627K
- A(H5N5) viruses demonstrated rapid 100% mortality and some transmission in ferrets
- A(H5N5) viruses preferred avian virus receptors and are sensitive to NA inhibitors
