# Supplementary material for: Multiple transatlantic incursions of highly pathogenic avian influenza clade 2.3.4.4b A(H5N5) virus into North America and spillover to mammals
Source: Cell Rep. Author manuscript; Available in PMC 2024 Aug 7. (PMC11305400; doi:10.1016/j.celrep.2024.114479)
Supplement: 1 [file NIHMS2011886-supplement-1.pdf]

Cell Reports, Volume *43*

Supplemental information

Multiple transatlantic incursions of highly pathogenic avian influenza clade 2.3.4.4b A(H5N5) virus into North America and spillover to mammals

Cassidy N.G. Erdelyan, Ahmed Kandeil, Anthony V. Signore, Megan E.B. Jones, Peter Vogel, Konstantin Andreev, Cathrine Arnason Bøe, Britt Gjerset, Tamiru N. Alkie, Carmencita Yason, Tamiko Hisanaga, Daniel Sullivan, Oliver Lung, Laura Bourque, Ifeoluwa Ayilara, Lemarie Pama, Trushar Jeevan, John Franks, Jeremy C. Jones, Jon P. Seiler, Lance Miller, Samira Mubareka, Richard J. Webby, and Yohannes Berhane

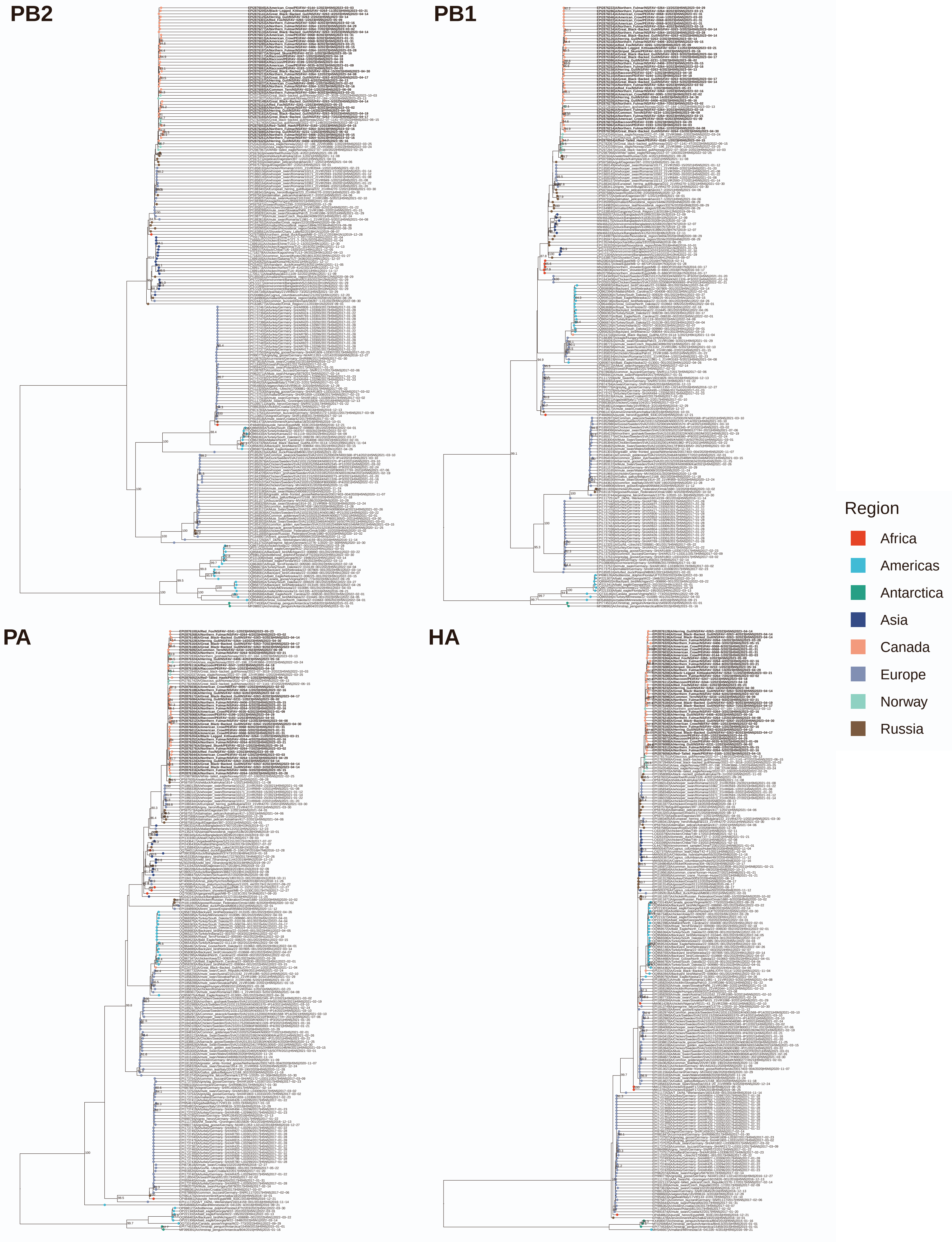
PB2
PB1
PA
HA
Region
Africa
Americas
Antarctica
Asia
Canada
Europe
Norway
Russia

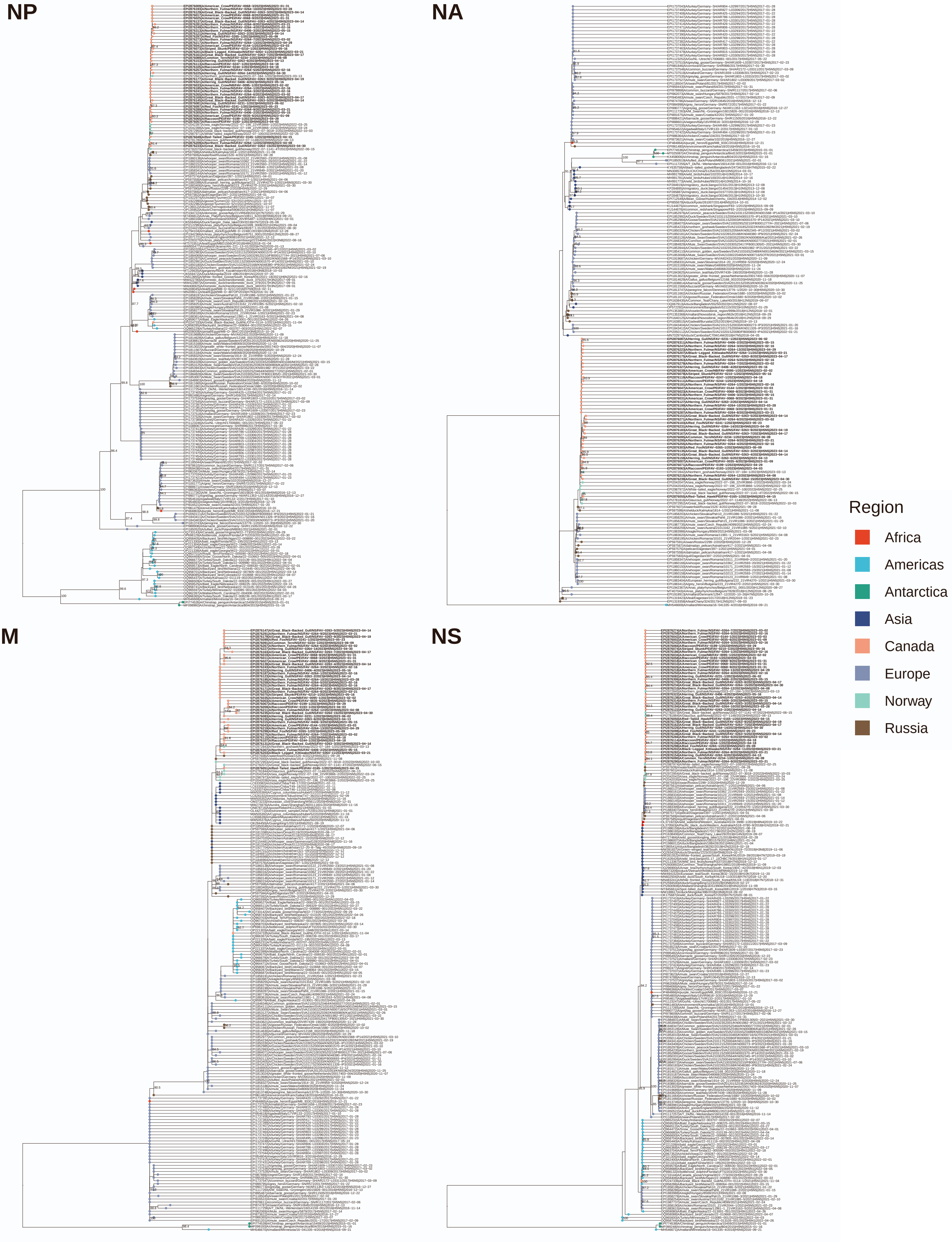
NP
NA
M
NS
Region
Africa
Americas
Antarctica
Asia
Canada
Europe
Norway
Russia

Figure S1. Maximum-likelihood phylogenetic trees of A(H5N5) viruses and reference A(H5N1) strains. Values are from 1000 replicates of the Shimodaira–Hasegawa approximate likelihood ratio test.

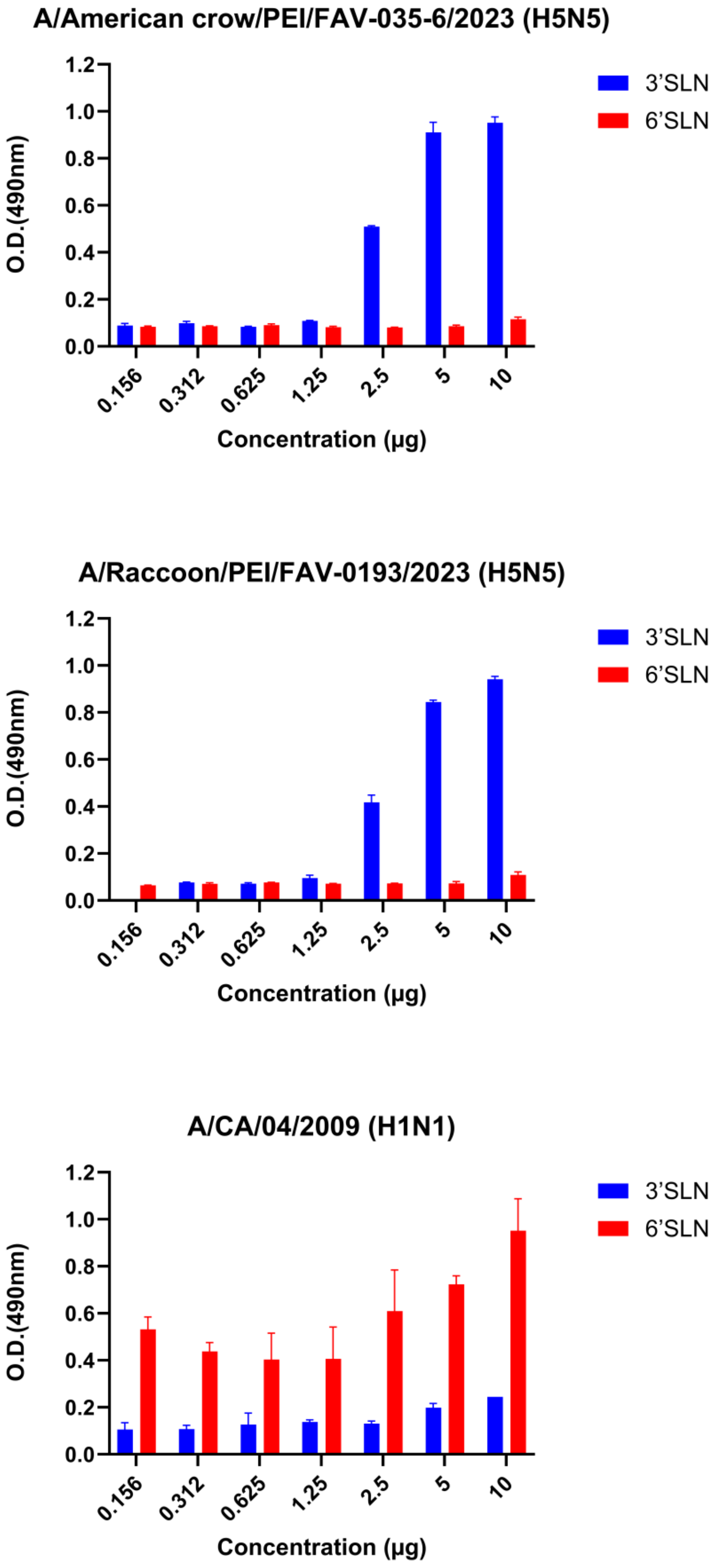

Figure S2. Solid-phase binding assay of A(H5N5) viruses to biotinylated sialylglycopolymers 3'SLN (avian virus preferred receptor; α-2,3) and 6'SLN (human virus preferred receptor; α-2,6). Influenza A/CA/04/2009 (H1N1) was included as a control for human preferred receptor viral binding. Data are shown as the mean ± SD from duplicate wells at each tested concentration.

Table S1. Amino acid substitutions with biologically significant effects described by Suttie*, et al.* [S1] that were present in Canadian A(H5N5) isolates.

Gene	Mutation	Function	Isolate	Ref
HA	S133A	Increased pseudovirus binding to α2–6	All	[S2]
HA	X154N	Increased virus binding to α2–6; **Required for glycosylation**	All	[S3]
HA	T156A	Increased virus binding to α2,6, increased transmission in guinea pigs; **destroys glycosylation sequon**	A/White-tailed_eagle/Norway/2022-07-100/2022, A/sea_eagle/Norway/2022-07-198_22VIR3866-2/2022, A/sea_eagle/Norway/2022-07-196_22VIR3866-1/2022, A/Great_black-backed_gull/Norway/2022-07-3018-2/2022	[S3,S4]
HA	V210I	Increased virus binding to α2,6	A/Herring_Gull/NS/FAV-0231-1/2023	[S5]
HA	K218Q with S223R	Increased virus binding to α2,3 and α2,6	All, except A/Northern_Fulmar/NS/FAV-0264-13/2023 (R218)	[S6]
PB2	T271A	Increased polymerase activity in avian and mammalian cells	A/Raccoon/PEI/FAV-0193-1/2023	[S7]
PB2	I292V	Increased polymerase activity in mammalian cell line, increased virulence in mice	A/Black-Legged_Kittiwake/NS/FAV-0264-11/2023	[S8]
PB2	K389R	Increased polymerase activity and replication in mammalian cell line	All	[S9]
PB2	A588V	Increased polymerase activity in and replication in avian and mammalian cell lines, increased virulence in mice	A/Great_Black-Backed_Gull/NS/FAV-0264-15/2023	[S10]
PB2	V598T/I	Increased polymerase activity and replication in mammalian cells, increased virulence in mice	All, except A/American_Crow/PEI/FAV-0068-3/2023, A/American_Crow/PEI/FAV-0068-8/2023, A/American_Crow/PEI/FAV-0068-9/2023 (M598)	[S9]
PB2	E627K	Enhanced polymerase activity, increased virulence in mice,	A/Great_Black-Backed_Gull/NS/FAV-0263-8/2023, A/Herring_Gull/NS/FAV-0263-6/2023, A/Raccoon/PEI/FAV-0199-1/2023, A/Striped_Skunk/PEI/FAV-0210-1/2023	[S1]

		contributes to airborne pathogenicity of IAVs in ferrets and contact transmission in guinea pigs. Decreases polymerase activity and replication in avian cell lines. Decreases virulence in chickens.		
PB1	D3V	Increased polymerase activity and viral replication in avian and mammalian cell lines	All	[S11]
PB1	N105S/T	Increased virulence in mice	(T105): A/Raccoon/PEI/FAV-0244-1/2023, A/Raccoon/PEI/FAV-0247-1/2023	[S12,S13]
PB1	D622G	Increased polymerase activity and virulence in mice	All	[S14]
PB1-F2	N66S	Enhanced replication, virulence and antiviral response in mice	A/Black-Legged_Kittiwake/NS/FAV-0264-11/2023	[S15,S16]
PA	S37A	Increased polymerase activity in mammalian cell line	All	[S17]
PA	N383D	Increased polymerase activity in mammalian and avian cell lines	All	[S18,S19]
PA	S224P + N383D	Increased polymerase activity and enhanced viral replication in duck and mouse cell lines, increased virulence in mice and ducks	A/Great_Black-Backed_Gull/NS/FAV-0263-5/2023	[S18,S19]
PA	N409S	Increased polymerase activity and replication in mammalian cell line	All	[S17]
PA	A515T	Decreased polymerase activity in mammalian cell line, decreased virulence in ducks	All	[S20]

M1	N30D	Increased virulence in mice	All	[S21]
M1	I43M	Increased virulence in mice, chickens and ducks	All	[S22]
M1	T215A	Increased virulence in mice	All	[S21]
NS1	P42S	Increased virulence and decreased antiviral response in mice	All	[S23]
NS1	L103F with I106M	Increased virulence in mice	All, except A/Great_black-backed_gull/Norway/2022-07-3018-2/2022 (L103)	[S24,S25]
NS1	I106M	Increased viral replication in mammalian cells virulence in mice	All	[S26]
NS1	C138F	Increased replication in mammalian cells, decreased interferon response	All	[S27]
NS1	V149A	Increased virulence and decreased interferon response in chickens	All	[S28]

Table S2. Bayes factor support and posterior probability of HA-156 residue mutation.

From	To	Bayes Factor	Posterior Probability
A	S	7.09	0.85
A	T	5.00	0.80
S	T	3.01	0.71

Table S3. NAI and CENI susceptibility of currently circulating North American HPAI A(H5N5) viruses in birds and mammals.

Influenza A virus	NA [a] / PA substitution	NAI, IC_{50} ± SD [nM] (**fold change**) [c]			CENI baloxavir [b], EC_{50} ± SD (nM) (**fold change**) [c]	
		Oseltamivir	Zanamivir	Peramivir	PRA	IRINA
A/Raccoon/PEI/FAV-0193-1/2023 (H5N5)	PA-T40A	0.33 ± 0.02 (**1**)	0.23 ± 0.05 (**1**)	0.08 ± 0.01 (**1**)	0.38 ± 0.04 (**2**)	0.31 ± 0.01 (**1**)
A/American_Crow/PEI/FAV-0035-6/2023 (H5N5)	PA-T40A	0.36 ± 0.03 (**2**)	0.24 ± 0.02 (**1**)	0.08 ± 0.01 (**1**)	0.45 ± 0.10 (**2**)	1.05 ± 0.03 (**2**)
Reference viruses						
A/purple heron/Egypt/MB 933C/2016 (H5N5)	–	n/d	n/d	n/d	0.32 ± 0.00 (**1**)	0.83 ± 0.01 (**1**)
A/bald eagle/Florida/W22-134OP/2022 (H5N1)	–	0.23 ± 0.18	0.24 ± 0.13	0.10 ± 0.01	0.23 ± 0.01	0.59 ± 0.11
A/Denmark/528/2009 (H1N1)pdm09	NA-H274Y	240.88 ± 10.36 (**1,047**)	0.40 ± 0.38 (**2**)	29.00 ± 2.63 (**290**)	n/a	n/a
rg-A/California/04/2009 (H1N1)pdm09	PA-I38T	n/a	n/a	n/a	23.24 ± 2.94 (**101**)	26.11 ± 1.56 (**44**)

All measurements have been done in triplicate to ensure statistical significance. IC_{50} and EC_{50} correspond to the 50% inhibitory and effective concentrations of NAI and CENI baloxavir, respectively. Mean values from three independent experiments are provided ± the standard deviation (SD).

n/a, not applicable; n/d, no data; –, amino acid substitution is not present; rg, recombinant virus.

[a] N2 numbering.

[b] Baloxavir acid, the active metabolite form of the pro-drug baloxavir marboxil

[c] compared to *A/bald eagle/Florida/W22-134OP/2022*

Table S4. Earliest index cases of North American clade 2.3.4.4 A(H5Nx) isolates compared to similar global isolates.

Clade	Location	Type	Date	Isolate
2.3.4.4c	Japan	H5N8	2014-11-23	A/crane/Kagoshima/KU1/2014
	Whatcom County, WA	H5N8	2014-12-08	A/gyrfalcon/Washington/41088-6/2014
2.3.4.4b	Netherlands	H5N1	2021-04-23	A/white-tailed eagle/Netherlands/21027616-001/2021
	St. John's, NL	H5N1	2021-11-04	A/Great_black-backed_gull/NL/OTH-0114-1/2021
	**Netherlands**	**H5N5**	**2016-12-13**	**A/M_Swan/NL-Groningen/16015826-001/2016**
	**Germany**	**H5N5**	**2016-12-13**	**A/swan/Germany-SN/R10645/2016**
	**Germany**	**H5N5**	**2020-10-29**	**A/buzzard/Germany-MV/AI02166/2020**
	Norway	H5N5	2022-02-25	A/White-tailed_eagle/Norway/2022−07−100/2022
	Summerside, PEI	H5N5	2023-01-09	A/American_Crow/PEI/FAV-0035-6/2023

References

1. Suttie, A.; Deng, Y.-M.; Greenhill, A.R.; Dussart, P.; Horwood, P.F.; Karlsson, E.A. Inventory of molecular markers affecting biological characteristics of avian influenza a viruses. *Virus Genes* **2019**, *55*, 739-768.
2. Yang, Z.-Y.; Wei, C.-J.; Kong, W.-P.; Wu, L.; Xu, L.; Smith, D.F.; Nabel, G.J. Immunization by avian h5 influenza hemagglutinin mutants with altered receptor binding specificity. **2007**, *317*, 825-828.
3. Wang, W.; Lu, B.; Zhou, H.; Suguitan, A.L.; Cheng, X.; Subbarao, K.; Kemble, G.; Jin, H. Glycosylation at 158n of the hemagglutinin protein and receptor binding specificity synergistically affect the antigenicity and immunogenicity of a live attenuated h5n1 a/vietnam/1203/2004 vaccine virus in ferrets. **2010**, *84*, 6570-6577.
4. Gao, Y.; Zhang, Y.; Shinya, K.; Deng, G.; Jiang, Y.; Li, Z.; Guan, Y.; Tian, G.; Li, Y.; Shi, J.*, et al.* Identification of amino acids in ha and pb2 critical for the transmission of h5n1 avian influenza viruses in a mammalian host. *PLOS Pathogens* **2009**, *5*, e1000709.
5. Watanabe, Y.; Ibrahim, M.S.; Ellakany, H.F.; Kawashita, N.; Mizuike, R.; Hiramatsu, H.; Sriwilaijaroen, N.; Takagi, T.; Suzuki, Y.; Ikuta, K. Acquisition of human-type receptor binding specificity by new h5n1 influenza virus sublineages during their emergence in birds in egypt. *PLOS Pathogens* **2011**, *7*, e1002068.
6. Guo, H.; de Vries, E.; McBride, R.; Dekkers, J.; Peng, W.; Bouwman, K.; Nycholat, C.; Verheije, M.H.; Paulson, J.; van Kuppeveld, F.J.M.*, et al.* Highly pathogenic influenza a(h5nx) viruses with altered h5 receptor-binding specificity. *Emerging Infectious Disease journal* **2017**, *23*, 220.
7. Bussey, K.A.; Bousse, T.L.; Desmet, E.A.; Kim, B.; Takimoto, T. Pb2 residue 271 plays a key role in enhanced polymerase activity of influenza a viruses in mammalian host cells. **2010**, *84*, 4395-4406.
8. Gao, W.; Zu, Z.; Liu, J.; Song, J.; Wang, X.; Wang, C.; Liu, L.; Tong, Q.; Wang, M.; Sun, H.*, et al.* Prevailing i292v pb2 mutation in avian influenza h9n2 virus increases viral polymerase function and attenuates ifn-β induction in human cells. **2019**, *100*, 1273-1281.
9. Hu, M.; Yuan, S.; Zhang, K.; Singh, K.; Ma, Q.; Zhou, J.; Chu, H.; Zheng, B.-J. Pb2 substitutions v598t/i increase the virulence of h7n9 influenza a virus in mammals. *Virology* **2017**, *501*, 92-101.
10. Xiao, C.; Ma, W.; Sun, N.; Huang, L.; Li, Y.; Zeng, Z.; Wen, Y.; Zhang, Z.; Li, H.; Li, Q.*, et al.* Pb2-588 v promotes the mammalian adaptation of h10n8, h7n9 and h9n2 avian influenza viruses. *Scientific Reports* **2016**, *6*, 19474.
11. Elgendy, E.M.; Arai, Y.; Kawashita, N.; Daidoji, T.; Takagi, T.; Ibrahim, M.S.; Nakaya, T.; Watanabe, Y. Identification of polymerase gene mutations that affect viral replication in h5n1 influenza viruses isolated from pigeons. **2017**, *98*, 6-17.
12. Taft, A.S.; Ozawa, M.; Fitch, A.; Depasse, J.V.; Halfmann, P.J.; Hill-Batorski, L.; Hatta, M.; Friedrich, T.C.; Lopes, T.J.S.; Maher, E.A.*, et al.* Identification of mammalian-adapting mutations in the polymerase complex of an avian h5n1 influenza virus. *Nature Communications* **2015**, *6*, 7491.
13. Song, M.-S.; Hee Baek, Y.; Kim, E.-H.; Park, S.-J.; Kim, S.; Lim, G.-J.; Kwon, H.-i.; Pascua, P.N.Q.; Decano, A.G.; Lee, B.-J.*, et al.* Increased virulence of neuraminidase inhibitor-resistant pandemic h1n1 virus in mice. *Virulence* **2013**, *4*, 489-493.
14. Feng, X.; Wang, Z.; Shi, J.; Deng, G.; Kong, H.; Tao, S.; Li, C.; Liu, L.; Guan, Y.; Chen, H. Glycine at position 622 in pb1 contributes to the virulence of h5n1 avian influenza virus in mice. **2016**, *90*, 1872-1879.
15. Schmolke, M.; Manicassamy, B.; Pena, L.; Sutton, T.; Hai, R.; Varga, Z.T.; Hale, B.G.; Steel, J.; Pérez, D.R.; García-Sastre, A. Differential contribution of pb1-f2 to the virulence of highly

pathogenic h5n1 influenza a virus in mammalian and avian species. *PLOS Pathogens* **2011**, *7*, e1002186.
16. Conenello, G.M.; Zamarin, D.; Perrone, L.A.; Tumpey, T.; Palese, P. A single mutation in the pb1-f2 of h5n1 (hk/97) and 1918 influenza a viruses contributes to increased virulence. *PLOS Pathogens* **2007**, *3*, e141.
17. Yamayoshi, S.; Yamada, S.; Fukuyama, S.; Murakami, S.; Zhao, D.; Uraki, R.; Watanabe, T.; Tomita, Y.; Macken, C.; Neumann, G.*, et al.* Virulence-affecting amino acid changes in the pa protein of h7n9 influenza a viruses. **2014**, *88*, 3127-3134.
18. Song, J.; Xu, J.; Shi, J.; Li, Y.; Chen, H. Synergistic effect of s224p and n383d substitutions in the pa of h5n1 avian influenza virus contributes to mammalian adaptation. *Scientific Reports* **2015**, *5*, 10510.
19. Song, J.; Feng, H.; Xu, J.; Zhao, D.; Shi, J.; Li, Y.; Deng, G.; Jiang, Y.; Li, X.; Zhu, P.*, et al.* The pa protein directly contributes to the virulence of h5n1 avian influenza viruses in domestic ducks. **2011**, *85*, 2180-2188.
20. Hulse-Post, D.J.; Franks, J.; Boyd, K.; Salomon, R.; Hoffmann, E.; Yen, H.L.; Webby, R.J.; Walker, D.; Nguyen, T.D.; Webster, R.G. Molecular changes in the polymerase genes (pa and pb1) associated with high pathogenicity of h5n1 influenza virus in mallard ducks. **2007**, *81*, 8515-8524.
21. Fan, S.; Deng, G.; Song, J.; Tian, G.; Suo, Y.; Jiang, Y.; Guan, Y.; Bu, Z.; Kawaoka, Y.; Chen, H. Two amino acid residues in the matrix protein m1 contribute to the virulence difference of h5n1 avian influenza viruses in mice. *Virology* **2009**, *384*, 28-32.
22. Nao, N.; Kajihara, M.; Manzoor, R.; Maruyama, J.; Yoshida, R.; Muramatsu, M.; Miyamoto, H.; Igarashi, M.; Eguchi, N.; Sato, M.*, et al.* A single amino acid in the m1 protein responsible for the different pathogenic potentials of h5n1 highly pathogenic avian influenza virus strains. *PLOS ONE* **2015**, *10*, e0137989.
23. Jiao, P.; Tian, G.; Li, Y.; Deng, G.; Jiang, Y.; Liu, C.; Liu, W.; Bu, Z.; Kawaoka, Y.; Chen, H. A single-amino-acid substitution in the ns1 protein changes the pathogenicity of h5n1 avian influenza viruses in mice. **2008**, *82*, 1146-1154.
24. Kuo, R.-L.; Krug, R.M. Influenza a virus polymerase is an integral component of the cpsf30-ns1a protein complex in infected cells. **2009**, *83*, 1611-1616.
25. Spesock, A.; Malur, M.; Hossain, M.J.; Chen, L.-M.; Njaa, B.L.; Davis, C.T.; Lipatov, A.S.; York, I.A.; Krug, R.M.; Donis, R.O. The virulence of 1997 h5n1 influenza viruses in the mouse model is increased by correcting a defect in their ns1 proteins. **2011**, *85*, 7048-7058.
26. Ayllon, J.; Domingues, P.; Rajsbaum, R.; Miorin, L.; Schmolke, M.; Hale, B.G.; García-Sastre, A. A single amino acid substitution in the novel h7n9 influenza a virus ns1 protein increases cpsf30 binding and virulence. **2014**, *88*, 12146-12151.
27. Li, J.; Zhang, K.; Chen, Q.; Zhang, X.; Sun, Y.; Bi, Y.; Zhang, S.; Gu, J.; Li, J.; Liu, D.*, et al.* Three amino acid substitutions in the ns1 protein change the virus replication of h5n1 influenza virus in human cells. *Virology* **2018**, *519*, 64-73.
28. Li, Z.; Jiang, Y.; Jiao, P.; Wang, A.; Zhao, F.; Tian, G.; Wang, X.; Yu, K.; Bu, Z.; Chen, H. The ns1 gene contributes to the virulence of h5n1 avian influenza viruses. **2006**, *80*, 11115-11123.
